# Comparative analysis of the mucosal and shell microbiota of *Trachemys scripta elegans* across multiple urban freshwater habitats

**DOI:** 10.1371/journal.pone.0353172

**Published:** 2026-07-10

**Authors:** Mariangel Correa Orellana, Coleman Odom, Anastasia Kuzmina, Jessica Urias-Quiroz, Laura Arias, Alyssa Kwiatowski, Georgina Aitolo, Patrick Martin, Colby Seitze, Meredith Winfield, Aspen Frisch, Jenna Tansiongco, Vishwa Venkatesan, Nadja La Point, Travis J. Laduc, Justin C. Havird

**Affiliations:** 1 Department of Integrative Biology, The University of Texas at Austin, Austin, Texas, United States of America; 2 College of Natural Sciences, The University of Texas at Austin, Austin, Texas, United States of America; 3 Department of Molecular Biosciences, The University of Texas at Austin, Austin, Texas, United States of America; Griffith University, AUSTRALIA

## Abstract

Turtles harbor diverse microbial communities that influence their health, ecology, and interactions with the environment. While sea turtle microbiomes have received growing attention, the microbial communities associated with freshwater turtles, particularly the widely distributed and invasive red-eared slider (*Trachemys scripta elegans*), remain understudied. Here, we used 16S rRNA gene sequencing to characterize the microbiomes of 42 red-eared sliders across four urban aquatic habitats in Austin, Texas, USA, sampling five body locations: carapace, plastron, skin, oral cavity, and cloaca. A total of 142 samples yielded 20,160 Amplicon Sequence Variants (ASVs), with community composition most strongly structured by body location, but also by geographic habitat. External surfaces (carapace, plastron, skin) were dominated by Cyanobacteria, Proteobacteria, and Deinococcota, while oral and cloacal samples exhibited higher proportions of Bacteroidota and site-specific variation. Alpha diversity differed significantly across habitats of origins but not across body locations, while beta diversity analyses revealed distinct microbial profiles among body regions. Notably, we report the first characterization of the oral microbiome in red-eared sliders, which was dominated by Proteobacteria and Deinococcota—patterns consistent with other reptiles. These findings shed light on microbial communities in invasive freshwater turtles and emphasize the need for broader microbial surveillance in urban aquatic ecosystems where wildlife and humans frequently interact.

## Introduction

Turtles host diverse microbial communities that play essential roles in their health, development, and ecological interactions [1,2]. While the microbiome of sea turtles has been thoroughly studied, revealing an incredible diversity of bacteria, archaea, and eukaryotic microorganisms [1–5], the microbiomes of freshwater turtles remain relatively poorly studied. However, freshwater turtles play significant ecological roles [6], can reach some of the highest documented levels of biomass per hectare among vertebrates, and are among the most threatened groups of vertebrates, with more than 50% of species endangered or recently extinct [7]. Freshwater turtles are also vectors of diseases, both those affecting humans and other animals [8–11]. A previous study of six freshwater turtle species in Georgia found that 45 out of 194 individuals tested positive for the potential human pathogen *Salmonella* [12]. Documenting the diversity of microbes living in and on freshwater turtles and what shapes this diversity may be important for ecological and public health.

Some studies have examined the gastrointestinal microbial communities of different freshwater turtle species, including semiaquatic three-toed turtles, which were dominated by Firmicutes and Bacteroidetes [13]. Microbial communities of the freshwater turtle *Mauremys reevesii* were also found to be affected more by ammonia compared to other freshwater turtles [14]. Red-eared sliders (*Trachemys scripta elegans*) are freshwater turtles native to the Mississippi River and its surrounding basin that represent a potentially powerful model for microbial ecology of freshwater turtles [15]. Due to international and domestic pet trade, red-eared sliders have become one of the most harmful invasive alien species worldwide [16–19]. Outside their native range, red-eared sliders compete with native species for food and habitat and can act as vectors for disease transmission [20,21]. In California, for instance, red-eared sliders have established populations in areas of the Sacramento River with high human traffic, indicating ongoing introductions [22]. High population densities of red-eared sliders in this area have been linked to negative impacts on the native western pond turtle (*Emys marmorata*), including population declines [23,24]. A similar situation has been observed in the Iberian Peninsula, where red-eared sliders directly compete with the native European pond turtle (*Emys orbicularis*), which is classified as Near Threatened, and the Spanish pond turtle (*Mauremys leprosa*), classified as Vulnerable [25,26].

Introduced red-eared sliders may also alter native environmental microbiomes [27,28] or microbiomes of native turtle species, potentially compromising their immune function, nutritional physiology, and disease resistance [29,30]. For example, invasive red-eared sliders in Mediterranean freshwater marshes can act as a chronic reservoir of fungal pathogens and serve as a vector for their transmission to native species [31]. Similarly, in China a highly pathogenic strain of *Salmonella* Pomona (*S. enterica* serovar Pomona) was isolated from wild invasive red-eared sliders—a strain known to pose serious health risks to humans, particularly children [30]. These findings suggest that understanding microbial ecology of red-eared sliders, particularly in urban environments, may be important for the conservation of native species and the prevention of human disease [8–10,32,33].

Despite being one of the world’s worst invasive species [34], the widespread introduction of red-eared sliders and their proximity to urban environments makes them ideal models to investigate the drivers of microbial diversity in freshwater turtles. However, few studies have examined microbial ecology in red-eared sliders. Different shell locations appear to harbor distinct communities, with less variation attributed to the specific habitat where a turtle was sampled, suggesting behavioral or physiological differences outweigh environmental differences between similar habitats in structuring microbial communities [7,35]. Supporting this, shell microbiomes were largely explained by basking behavior and species identity among six freshwater turtles, not collection site [36]. However, these studies focused on shell microbiomes and undeveloped habitats, and it is unknown whether their findings extend to internal microbiomes or urban environments.

Here, we examined red-eared sliders’ microbiomes in mucosal surfaces (oral and cloaca) and the plastron and carapace in turtles from urban environments in the Austin, Texas, USA metro area. We aimed to more thoroughly describe microbial communities in freshwater turtles and hypothesized that variation in microbiomes would be explained more by body location sampled than habitat of origin, following the results of shell microbiome studies in more natural habitats [7,35,37]. We also sought to leverage the ubiquity of red-eared sliders in urban settings as a training system to involve undergraduate students at the University of Texas at Austin (UT) in biodiversity research, enabling them to gain hands-on research experience in field sampling efforts and data analysis. Our study provides insights into the microbiome of red-eared sliders in nearby urban habitats.

## Methods

### Site descriptions and sampling

**Fig 1:**
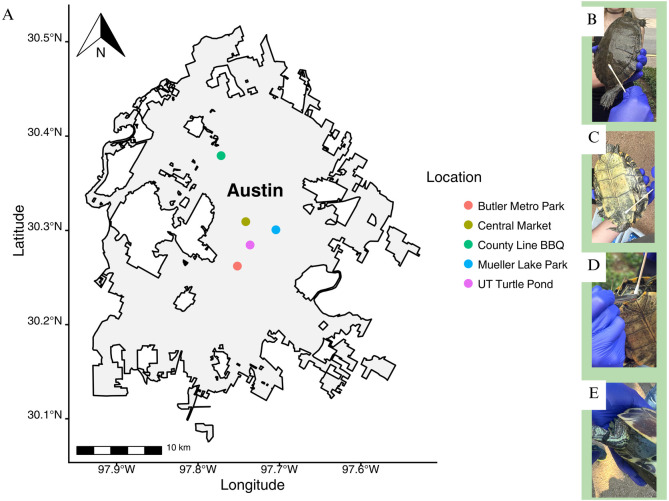
Geographic distribution of turtle sampling sites. (A) Map of sampling locations for turtles across Austin, Texas, USA. The right panel includes insets illustrating the specific body sites sampled: (B) carapace, (C) plastron, (D) oral cavity, and (E) leg (skin). Map boundary and water data obtained from the U.S. Census Bureau TIGER/Line Shapefiles via the R tigris package (public domain;https://www.census.gov/geographies/mapping-files/time-series/geo/tiger-line-file.html).

Samples were collected from five locations in Austin, Texas, USA (Fig 1A), between September 2023 and May 2024 (Table S1 in S1 File). The first site was the UT Turtle Pond, a ~ 1,541 m^2^ pond on the UT campus, which was home to ~100 turtles at the time of sampling, primarily red-eared sliders. This site was sampled twice during the study (11 October and 01 November), collecting seventy samples from fourteen individuals, 13 red-eared sliders, and 1 Texas river Cooter. The second site, Butler Metro Park Pond in Downtown Austin (~2,786 m^2^), yielded fifty samples from ten individuals, all sampled on 29 March 2024 and all of which were red-eared sliders. Similarly, another ten individual turtles were sampled from a ~ 1,997 m^2^ pond in Hyde Park Community Central Park, all of which were red-eared sliders, yielding 50 samples collected on 11 April 2024. These first three locations were largely similar habitats in terms of their apparent biotic diversity, sizes, and flow regimes. The third site was Lake Mueller, a much larger 25,090 m^2^ lake, where two red-eared sliders were sampled on 19 April 2024. Only two individuals were sampled at this location due to technical constraints. Lastly, we collected samples from behind the County Line on the Lake restaurant, positioned along Bull Creek, a tributary of the Colorado River, where an additional ten turtles were sampled (9 red-eared sliders, 1 Texas river Cooter), yielding 50 samples on 6 May 2024. This last site was noticeably different, mostly because of the significant flow of water during the time of sampling.

Turtles were caught by hand or with dip nets and then carefully handled with nitrile gloves. A 6-inch sterile DNA-free standard cotton swab with a wooden handle (Puritan, USA) was used to collect samples via ~30s of vigorous swabbing from five body locations: carapace (lower right pleural and anal scutes, Fig 1B), plastron (lower left femoral and anal scutes, Fig 1C), oral cavity (including cheeks, tongue, and throat, Fig 1D), skin (inner thigh of right hind-leg, Fig 1E), and cloacal cavities (up to ~1 inch). Immediately after sampling, swabs were placed in ~2 mL of DNA/RNA Shield (Zymo, USA) and then stored at −20 ºC no more than three hours after sampling. Sampled turtles were PIT-tagged using BioMark HDX12 tags in the inner thigh of the right hind leg (sealed with “skin glue”), except some turtles at the Bull Creek site that had already been PIT-tagged. Turtles were also photographed, weighed to the nearest 0.001 kg, and measured (length, width, and height) before releasing at the site of capture.

The UT Institutional Animal Care and Use Committee and Institutional Biosafety Committee approved all turtle capture methods and field practices (IACUC protocol # AUP-2023–00091 and IBC protocol # IBC-2023–00133). Upon release, all turtles were monitored for normal swimming behavior for 5 minutes or until they disappeared, and turtles were normally handled for < 10 minutes during sampling before release. For sampling conducted on private property (County Line BBQ), permission was obtained from landowners prior to fieldwork.

### DNA extractions and sequencing

DNA was extracted from samples no more than one month after collection using the Qiagen DNeasy PowerSoil Pro Kit (Hilden, Germany), following the manufacturer’s recommendations. The Qiagen vortex adapter was used for cell lysis, and the samples were vortexed for 10 minutes. Following extraction, the concentration of the resulting DNA was assessed using the Invitrogen Qubit™ dsDNA Broad Range and/or High-Sensitivity quantification kits (Fisher Scientific, UK) on an Invitrogen™ Qubit™ 4 fluorometer (Fisher Scientific, UK). DNA concentrations ranged from 0.5 to 120 ng/μl, but were normalized to 10 ng/μl prior to submission to the Genomic Sequencing and Analysis Facility (GSAF) at UT.

In total, 142 samples that passed quality control steps were used for PCR and library preparation using 20 ng of DNA per sample, with the primer set Pro341F/Pro805R [35,38] to amplify the V3–V4 regions of the 16S rRNA gene. This approach was used to produce prokaryote-biased sequence reads. The samples were then sequenced on a single flow cell of an Illumina NextSeq 1000, yielding 300 bp paired end reads, with a target of ~400,000 reads per sample.

### Microbiome composition analyses

Demultiplexed sequence reads were delivered by the UT GSAF and imported into the Quantitative Insights into Microbial Ecology 2 (QIIME2) [39] Amplicon 2024 pipeline. Primers and adapters were removed from both forward and reverse reads using the QIIME2 trimming feature, which employs cutadapt [40]. Denoising was performed using the QIIME2 DADA2 plugin, with forward and reverse reads trimmed to 276 and 180 bases, respectively, due to a decline in read quality beyond those positions [41]. Amplicon sequence variants (ASVs) were inferred from raw sequencing reads using DADA2.The resulting table was filtered to retain ASVs with a minimum frequency of 5 sample and present in at least 5 (of 142) samples. We employed this strategy to target relatively common ASVs, but acknowledge that it likely misses rare ASVs that might be specific to combinations of body locations and sites. Taxonomy was assigned using the q2-feature-classifier plugin with the SILVA v132 classifier [42]. Taxonomic composition was visualized at the phylum level (level 2 in QIIME) using the QIIME2 taxa bar plot plugin and the QIIME2View online tool. A phylogenetic tree was constructed using the QIIME2 phylogeny plugin for downstream alpha and beta diversity analyses [43,44]. Rarefaction curves were generated, and the maximum sampling depth was set to 150,000 based on post-DADA2 read counts to retain the majority of samples.

### Microbial diversity and statistical analyses

Diversity analyses were performed using the QIIME2 diversity core-metrics-phylogenetic plugin [45]. The resulting rooted phylogenetic tree was used to calculate the alpha and beta diversity of the samples, which were compared with the collected samples’ habitat of origin (five sites described in Fig 1) and body location (plastron, cloaca, oral, skin, carapace). Faith Phylogenetic Diversity and Evenness analyses [45,46] were used to analyze alpha diversity. Bray-Curtis dissimilarity indices were exported and analyzed in R using the vegan package [47] for the beta diversity and tested for statistical differences between the body locations, habitats of origin,species, and sex using PERMANOVA (see below).

To visualize community dissimilarity patterns among samples, non-metric multidimensional scaling (NMDS) [48] was performed on the Bray-Curtis dissimilarity matrix derived from quantitative features [49]. First, the Bray-Curtis dissimilarity matrix and corresponding metadata were imported into R using the read.delim function. The Bray-Curtis matrix and metadata were merged using the merge function and dissimilar indices among samples were computed using the vegdist function. NMDS was then performed with the metaMDS function, specifying three dimensions (k = 3), to reduce the community dissimilarity structure to a lower-dimensional space.

Sample metadata variables (i.e., body location, habitat of origin, and sex) were standardized using the str_to_title function from the stringr package to ensure consistent formatting. The resulting NMDS scores were extracted using the scores function and combined with metadata for plotting. Finally, an NMDS ordination plot was generated using ggplot2, where points were colored by body location and shaped by habitat of origin to visualize clustering patterns among samples.

Single, multivariate PERMANOVA tests [50] were performed using the adonis2 function in the vegan package to statistically assess whether microbial community composition differed significantly by body location, habitat of origin, and/or sex. The test was based on the Bray-Curtis dissimilarity matrix, and significance was determined using 999 permutations.

## Results

### Cyanobacteria and Deinococcota dominate external body sites

In total, 110,810,584 sequence reads were generated across all samples (publicly available via NCBI’s SRA database under Project ID PRJNA1331802), including both forward and reverse reads. On average, 779,776 ± 39,289 (SEM) reads were generated per sample, largely exceeding our target (Table S1 in S1 File). A total of 341,049 ASVs were initially detected across all samples. After primer trimming, denoising, and filtering for relatively common ASVs, a total of 20,160 ASVs were identified across samples and retained for downstream analyses. All assigned sequence variants had a relatively high confidence (>90%).

**Fig 2:**
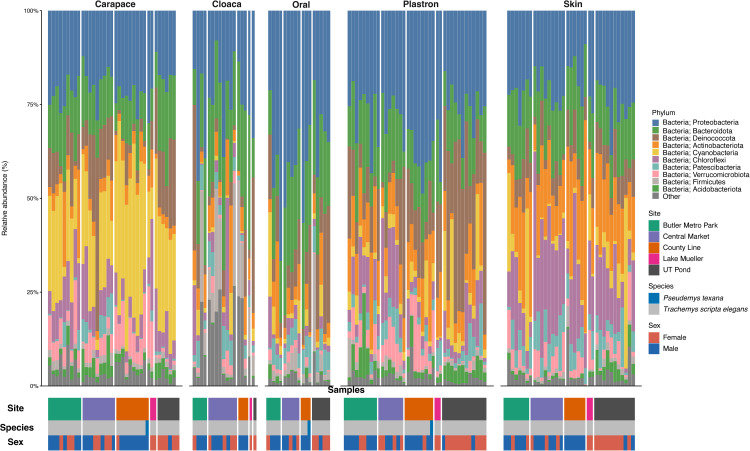
Microbiome 16S rRNA profiles across body locations and habitats from red-eared sliders based on the V3-V4 region. Samples are grouped according to body location, which explained the most variation in microbial diversity, and then by habitat of origin. T refers to the 2 Texas river cooters included in the study (*Pseudemys texana*).

Taxonomic assignment of ASVs was high, with <10% of common ASVs (found in at least 5 samples) being unassigned. Although generally diverse (Fig 2), in several cases, carapace, plastron, and skin samples were dominated by Cyanobacteria (exceeding 50% relative abundance), followed by Proteobacteria (≥30%) and Deinococcota (≥20%). Cloacal samples exhibited more variation across different habitats. Notably, samples from County Line had a higher relative abundance of Fusobacteria (>60%) than those from the urban ponds (~10%). Cloacal samples also included notable proportions of Bacteroidota (≥20%) and Proteobacteria (>10%). Oral samples were primarily composed of Bacteroidota (>20%), Proteobacteria (≥20%), and Deinococcota, which reached up to 65% in some samples. When ASVs were not filtered to include only those found in at least 5 samples, with less than 10% of ASVs generally being unassigned (Fig. S1 in S1 File).

Across samples within Cyanobacteria, *Synechoccus* was the most abundant genus (>50%) and the second most abundant was *Xenocccacea* (>20%). For the Deinococcota, the most abundant genus was *Deinoccocus* (>40%) and for Proteobacteria the most abundant class was Gammaproteobacteria (≥30%). For the cloacal samples, the most abundant Fusobacteria was *Cetobacterium*.

### Microbial diversity varies most by body location

Despite high initial sequencing depth (~700,000 reads per sample), DADA2 processing (quality filtering, and denoising) reduced the number of usable reads per sample. Alpha rarefaction curves plateaued at high sequencing depths (100,00–150,000 reads), indicating that the diversity in most samples was adequately captured with our sequencing efforts (Fig. S2 in S1 File). Although samples from the carapace and plastron exhibited the highest number of ASVs (averaging 2,886 in carapace and 2,130 ASVs in plastron samples, respectively), there were no statistically significant differences in alpha diversity evenness among body location (Kruskal-Wallis H = 6.48, *p* = 0.165). Howeverm significant differences in alpha diversity were observed among habitats (H = 10.51, *p* = 0.032), with samples from County Line and Central Market having the most ASVs (Fig. S2, S3 in S1 File).

Faith’s Phylogenetic Diversity varied significantly across body location (H = 13.95, *p* = 0.007) and habitat of origin (H = 13.70, *p* = 0.008). The carapace and cloacal samples had the highest phylogenetic diversity based on body location, while Central Market and County Line had higher diversity based on habitat of origin (Fig. S3 in S1 File).

**Fig 3:**
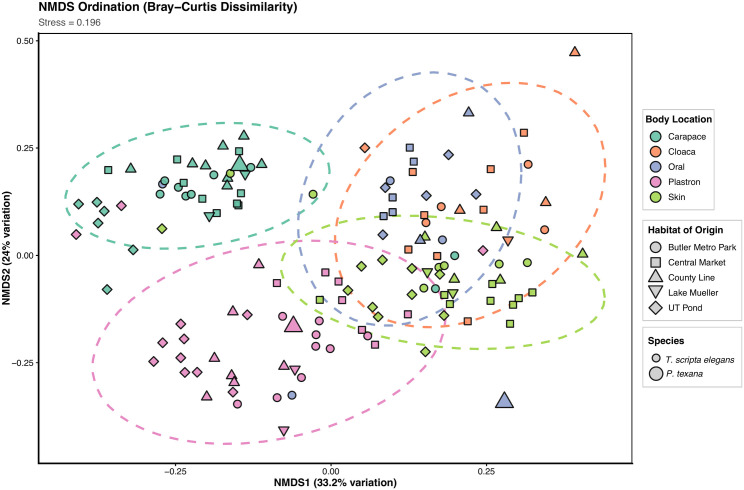
Non-metric multidimensional scaling (NMDS) ordinations of Bray Curtis beta diversity, visualizing dissimilarities in microbial communities across samples (stress = 0.196). Samples are colored by body location and shapes represent habitat of origin. Ellipses show 95% confidence intervals for body locations.

Beta diversity analysis using NMDS (Bray-Curtis dissimilarity, stress = 0.196) revealed distinct clustering of samples according to body location, with carapace and plastron samples having distinct communities, while those from oral and cloacal samples were more similar (Fig 3, Fig. S2 in S1 File). While there was some clustering according to habitat of origin, the patterns were not as distinct. Qualitatively similar results were found using binary Jaccard index (Fig. S4 in S1 File). A multivariate PERMANOVA based on Bray-Curtis dissimilarity confirmed significant differences in microbial composition by both body location (F = 11.86, R² = 0.23, *p* = 0.001) and habitat of origin (F = 6.09, R² = 0.11, *p* = 0.001), with these factors together explaining 35.2% of the observed variation. Sex did not explain a significant portion of variation after accounting for body location and habitat of origin (F = 1.32, R² = 0.006, *p* = 0.142, Fig. S5 in S1 File). PERMDISP analyses indicated no significant differences in multivariate dispersion among groups (all *p* > 0.05), supporting that observed differences reflect true compositional shifts. We also tested whether the two different species in our dataset had different communities, but species did not explain a significant portion of variation in microbial community composition (univariate PERMANOVA, R² = 0.003, *p* = 0.920). This result likely reflects the highly unbalanced sampling design, with only three samples from *Pseudemys texana*, limiting statistical power for species-level comparisons.

## Discussion

### Key microbial players in urban red-eared sliders

An organism’s microbiome is a potential source for disease, but also plays a vital role in maintaining host health [51,52]. Investigating the microbiome of freshwater turtles, especially invasive red-eared sliders, is essential for understanding whether turtles carry potential pathogens, if native turtle populations are healthy, and whether their microbial communities vary depending on ecological or physiological factors.

Here, we expanded on the limited number of studies examining microbiomes of freshwater turtles by focusing on microbial communities across body locations in red-eared sliders from urban environments. We find that certain bacterial taxa may be part of a stable, resident microbiome, while others are transient and may reflect environmental exposure. Taxonomic composition was generally consistent within body locations; for example, the shell microbiome (carapace and plastron) was dominated by Proteobacteria, Bacteroidota, and Cyanobacteria across different habitats (Fig 2, Fig. S1 in S1 File). This finding is similar to previous results in a study conducted on red-eared sliders in rivers of Oklahoma from a less urban setting [35]. However, our samples showed a higher relative abundance of Cyanobacteria, which suggests that the urban ponds sampled here may have high abundances of environmental Cyanobacteria, However, we did not collect environmental samples or sample across times/seasons. These limitations make it difficult to determine whether shell microbiomes are more similar to environmental microbiomes or might vary more across seasons. Notably, increased environmental Cyanobacteria abundance can lead to harmful algal blooms that present potential ecological concerns and might be harmful for resident turtles [53]. Cyanobacteria, including toxin-producing genera, have also been found in shells of loggerhead sea turtles [3] suggesting that turtles’ shells can be reservoirs of cyanobacteria. Notably, our study is among the first to characterize the oral microbiome of red-eared sliders, which was dominated by Bacteroidota, Proteobacteria, and Deinococcota—a pattern consistent with other reptiles [54].

Firmicutes were also recovered from cloacal samples, particularly the genus *Romboutsia*, which is commonly found in the gastrointestinal tract of vertebrates and is typically associated with healthy individuals [55]. We also identified the genus *Epulonipiscium*, a group of large, heterotrophic bacteria known to form symbiotic relationships with herbivorous surgeonfish that consume algae and detritus [56]. Red-eared sliders are opportunistic omnivores that consume a variety of food sources [57], suggesting the potential for mutualistic relationships with diverse bacterial genera that aid in digestion and nutrient absorption. However, no studies to date have specifically investigated these associations in red-eared sliders..

Among the bacteria recovered from our samples, we detected genera known to include human pathogens, including *Acinetobacter* [58], *Mycobacterium* [59], and *Roseomonas* [60]. We also identified *Erysipelothrix*, a genus known to be pathogenic to domestic pigs, poultry, and other animals [61]. Although we did not assess the pathogenic potential of these bacteria, these genera were found on both the carapace and plastron, suggesting incidental handling of turtles by the public could result in transmission. Moreover, our filtering methods and reliance on 16S profiling may have missed pathogens at low abundances. In a study conducted in the river streams of Oklahoma [7,35], researchers identified ten genera and two families of pathogenic bacteria, including *Enterococcus* (Enterococcaceae) and *Francisella* (Francisellaceae), both of which were also identified in our study. Previous studies conducted around the world [8,31,62–64] have demonstrated that there are disease-causing microbes in red-eared sliders, some of which are resistant to antibiotics. For example, *Salmonella pomella* and *Salmonella enterica* were isolated from wild red-eared sliders in China [8,62]. The invasion of urban areas by red-eared sliders may present a potential risk factor to public health, particularly in light of guidelines from agencies such as the U.S. Centers for Disease Control and prevention (CDC), which highlight the zoonotic risks associated with handling reptiles and amphibians. Notably, these taxa were not highly abundant in our study, suggesting that urban turtles may serve as incidental vectors rather than primary reservoirs of these microbes. Furthermore, 16S rRNA sequencing cannot directly assess for pathogenicity; therefore, additional research is necessary to determine the presence and potential risk of pathogenic organisms.

### Urban freshwater turtles harbor unique microbiomes across body locations and habitats

The observed differences in microbial community composition were largely dependent on the body location sampled (Fig 3), with less variation linked to the habitat of origin, and almost none linked to sex (Fig. S5 in S1 File). However, alpha diversity did not show statistically significant differences across body location, but were significantly different based on the habitat of origin, suggesting that geographic and environmental factors may play a more prominent role in shaping how many species are present, but not overall community composition. Moreover, the functional roles of microbes likely vary widely across body locations, with oral and cloacal samples being more indicative of the gut microbiome and shell microbiomes more linked to the environment. In our study, we did not include environmental water samples, so we were unable to directly compare environmental microbiomes to turtle microbiomes. However, given the different biotic and abiotic factors (especially flow rates) across habitats, many rarer microbial species may be transient recruits from the environment. Supporting this, a 2023 meta-analysis [65] of reptile gut microbiomes found that, in most cases, environmental factors were the primary drivers of alpha diversity.

Within samples from the same body location, microbial communities from turtles from the same habitat were often more similar to each other than those from other habitats (Fig 3). For example, carapace communities from the UT Turtle Pond formed a distinct cluster relative to other habitats, with UT turtles having carapace communities more similar to plastron communities from the same habitat than to carapace communities from other locations. However, limited sampling at Lake Mueller restricted our ability to make robust population-level inferences for this habitat.. Taken together, our results indicate body location is the most significant driver of microbial diversity patterns in freshwater turtles, although environmental conditions and habitat of origin also explain a significant amount of variation. Further sampling across seasons may further explain the drivers of microbial communities of red-eared slider. Whole-genome sequencing or culture-based approaches would also be useful to metabolically characterize turtle microbiomes.

### Urban ponds and red-eared sliders as natural biodiversity laboratories

Urban ponds are compelling environments for studying microbial diversity and biodiversity generally. Although previous studies have shown that urban ponds are generally less diverse than rural ponds, they still support a wide range of aquatic organisms and can play an important role in biodiversity conservation [66,67]. These ponds can also function as nature-based solutions in urban planning [68]. Many urban ponds vary in terms of light availability, nutrient levels, water sources, and exposure to anthropogenic activities, including sewage and stormwater runoff. These factors make them particularly interesting for investigating environmentally dependent organismal interactions. For example, we recovered the genus *Syntrophorhabdus*, which includes some strains known to have syntrophic associations with methanogens able to degrade phenol and isophthalate (3-carboxybenzoate), chemical compounds that have accumulated for many years in the atmosphere and cause negative effects on the environment [69,70]. The accessibility of urban ponds provides an opportunity to investigate such findings more thoroughly. Future directions for examining microbial ecology of freshwater turtles could make use of urban ponds by examining how microbial communities change with seasons, development [71,72], and whether communities are specific to individuals.

Importantly, our study was able to involve dozens of undergraduate researchers from UT. This was accomplished partly due to the popularity of the UT turtle pond as a landmark for appreciating nature in a very urban campus. We also intentionally set almost no barriers to students becoming involved in this research – everyone was welcome regardless of previous experience, whether they could participate long-term or just briefly, or their educational or career goals. Field experiences are important educational opportunities that can shape the career of aspiring ecologists more than experiences in a traditional classroom [73–76]. However, curating field experiences can be difficult due to accessibility, with many students from traditionally marginalized backgrounds missing out on these opportunities [74,75,77]. Many university and educational campuses in the US feature small freshwater ponds, many of which contain red-eared sliders. Our work further illustrates how these ponds represent an untapped resource to provide field experiences for students and conduct useful biodiversity-related research.

## Supporting information

S1 File Supplementary Figures and tables. Contains supplementary figures and tables referenced in the main text, including summary of sampling, number of reads, alpha rarefaction curves and unfiltered taxonomy and NMDS ordination plots.
(DOCX)
