# Supplementary material for: Comparative analysis of the mucosal and shell microbiota of Trachemys scripta elegans across multiple urban freshwater habitats
Source: PLoS One. 2026 Jul 10;21(7):e0353172. doi: 10.1371/journal.pone.0353172 (PMC13353938; doi:10.1371/journal.pone.0353172)
Supplement: S1 File — Contains supplementary figures and tables referenced in the main text, including summary of sampling, number of reads, alpha rarefaction curves and unfiltered taxonomy and NMDS ordination plots. (DOCX) [file pone.0353172.s001.docx]

Table S1. Summary of sampling dates, locations, number of turtles sampled, and the number of samples collected.

| **Site Name** | **Location** | **Sampling Date(s)** | **Sample Type** | **Number of**  **Turtles** | **Number of Samples** |
| --- | --- | --- | --- | --- | --- |
| UT Turtle Pond | Austin, Texas, USA | 10/11/2023 and 11/01/2023 | oral, cloaca, skin, carapace, plastron | 14 | 70 |
| Butler Metro Park | Austin, Texas, USA | 03/29/2024 | oral, cloaca, skin, carapace, plastron | 10 | 50 |
| Hyde Park Community Central Park | Austin, Texas, USA | 04/11/2024 | oral, cloaca, skin, carapace, plastron | 10 | 50 |
| Lake Mueller | Austin, Texas, USA | 04/19/2024 | cloaca, skin, carapace, plastron | 2 | 18 |
| County Line  BBQ | Austin,  Texas, USA | 05/06/2024 | oral, cloaca, skin, carapace, plastron | 10 | 50 |

Table S2. Number of raw paired end (PE) reads sequenced and amplified sequence variants (ASVs) for each of the microbial samples analyzed here, along with metadata on body location, habitat of origin, and sex for each sample.

| Sample | Body Location | Sex | Species | Habitat of Origin | Sampling Date | Number of PE reads | Unfiltered ASVs | Filtered ASVs |
| --- | --- | --- | --- | --- | --- | --- | --- | --- |
| A1_16C | Carapace | Male | Red-eared Slider (*Trachemys scripta elegans*) | Butler Metro Park | 03/29/2024 | 285803 | 4,248 | 2,041 |
| A1_37P | Plastron | Female | Red-eared Slider (*Trachemys scripta elegans)* | Lake Mueller | 04/19/2024 | 1025705 | 3,449 | 1,858 |
| A10_47C | Carapace | Female | Red-eared Slider (*Trachemys scripta elegans*) | County Line BBQ | 05/06/2024 | 932937 | 15,752 | 5,543 |
| A11_40P | Plastron | Male | Red-eared Slider (*Trachemys scripta elegans*) | County Line BBQ | 05/06/2024 | 439750 | 2,867 | 911 |
| A12_39S | Skin | Male | Red-eared Slider (*Trachemys scripta elegans*) | County Line BBQ | 05/06/2024 | 195746 | 2,339 | 931 |
| A2_23CO | Cloaca | Female | Red-eared Slider (*Trachemys scripta elegans*) | Butler Metro Park | 03/29/2024 | 612562 | 2,055 | 1,129 |
| A2_8C | Carapace | Female | Red-eared Slider (*Trachemys scripta elegans*) | UT Pond | 10/11/2023 | 866729 | 4,632 | 2,694 |
| A3_16P | Plastron | Male | Red-eared Slider (*Trachemys scripta elegans*) | Butler Metro Park | 03/29/2024 | 669001 | 3,457 | 1,603 |
| A3_2P | Plastron | Male | Texas Cooter (*Pseudemys texana*) | UT Pond | 10/11/2023 | 816649 | 3,350 | 1,633 |
| A4_22S | Skin | Male | Red-eared Slider (*Trachemys scripta elegans*) | Butler Metro Park | 03/29/2024 | 549476 | 7,610 | 3,505 |
| A4_3S | Skin | Female | Red-eared Slider (*Trachemys scripta elegans*) | UT Pond | 10/11/2023 | 472436 | 2,477 | 1,321 |
| A5_14S | Skin | Female | Red-eared Slider (*Trachemys scripta elegans*) | UT Pond | 11/1/2023 | 630026 | 2,773 | 1,340 |
| A5_32C | Carapace | Male | Red-eared Slider (*Trachemys scripta elegans*) | Central Market | 04/11/2024 | 647243 | 2,767 | 1,359 |
| A6_16S | Skin | Male | Red-eared Slider (*Trachemys scripta elegans*) | Butler Metro Park | 03/29/2024 | 406887 | 3,521 | 1,807 |
| A6_30CO | Cloaca | Female | Red-eared Slider (*Trachemys scripta elegans*) | Central Market | 04/11/2024 | 269004 | 2,165 | 804 |
| A7_30P | Plastron | Female | Red-eared Slider (*Trachemys scripta elegans*) | Central Market | 04/11/2024 | 206635 | 2,246 | 1,031 |
| A7_5P | Plastron | Female | Red-eared Slider (*Trachemys scripta elegans*) | UT Pond | 10/11/2023 | 679098 | 3,577 | 1,995 |
| A8_29S | Skin | Female | Red-eared Slider (*Trachemys scripta elegans*) | Central Market | 04/11/2024 | 278487 | 2,203 | 965 |
| A9_39C | Carapace | Male | Red-eared Slider (*Trachemys scripta elegans*) | County Line BBQ | 05/06/2024 | 296330 | 4,592 | 2,285 |
| B1_17C | Carapace | Male | Red-eared Slider (*Trachemys scripta elegans*) | Butler Metro Park | 03/29/2024 | 252059 | 3,528 | 1,579 |
| B1_36S | Skin | Female | Red-eared Slider (*Trachemys scripta elegans*) | Lake Mueller | 04/19/2024 | 996414 | 5,247 | 2,469 |
| B10_39CO | Cloaca | Male | Red-eared Slider (*Trachemys scripta elegans*) | County Line BBQ | 05/06/2024 | 933024 | 1,138 | 497 |
| B11_41P | Plastron | Male | Texas Cooter (*Pseudemys texana*) | County Line BBQ | 05/06/2024 | 1416666 | 10,686 | 5,095 |
| B12_40S | Skin | Male | Red-eared Slider (*Trachemys scripta elegans*) | County Line BBQ | 05/06/2024 | 408855 | 1,918 | 700 |
| B2_24CO | Cloaca | Male | Red-eared Slider (*Trachemys scripta elegans*) | Butler Metro Park | 03/29/2024 | 1469924 | 3,722 | 2,393 |
| B2_9C | Carapace | Female | Red-eared Slider (*Trachemys scripta elegans*) | UT Pond | 10/11/2023 | 1025413 | 5,764 | 3,416 |
| B3_17P | Plastron | Male | Red-eared Slider (*Trachemys scripta elegans*) | Butler Metro Park | 03/29/2024 | 487691 | 2,765 | 1,135 |
| B3_3P | Plastron | Female | Red-eared Slider (*Trachemys scripta elegans*) | UT Pond | 10/11/2023 | 745854 | 3,539 | 2,047 |
| B4_23S | Skin | Female | Red-eared Slider (*Trachemys scripta elegans*) | Butler Metro Park | 03/29/2024 | 766340 | 2,790 | 1,335 |
| B4_4S | Skin | Female | Red-eared Slider (*Trachemys scripta elegans*) | UT Pond | 10/11/2023 | 1038589 | 4,504 | 2,489 |
| B5_33C | Carapace | Male | Red-eared Slider (*Trachemys scripta elegans*) | Central Market | 04/11/2024 | 584553 | 9,785 | 3,416 |
| B5_33CO | Cloaca | Male | Red-eared Slider (*Trachemys scripta elegans*) | Central Market | 04/11/2024 | 641099 | 5,580 | 2,883 |
| B6_32CO | Cloaca | Male | Red-eared Slider (*Trachemys scripta elegans*) | Central Market | 04/11/2024 | 394287 | 1,534 | 569 |
| B6_43CO | Cloaca | Male | Red-eared Slider (*Trachemys scripta elegans*) | County Line BBQ | 05/06/2024 | 467346 | 1,841 | 911 |
| B9_40C | Carapace | Male | Red-eared Slider (*Trachemys scripta elegans*) | County Line BBQ | 05/06/2024 | 834912 | 11,580 | 4,459 |
| C1_19C | Carapace | Male | Red-eared Slider (*Trachemys scripta elegans*) | Butler Metro Park | 03/29/2024 | 697558 | 6,061 | 2,899 |
| C1_37S | Skin | Female | Red-eared Slider (*Trachemys scripta elegans*) | Lake Mueller | 04/19/2024 | 697558 | 3,335 | 1,941 |
| C10_42CO | Cloaca | Male | Red-eared Slider (*Trachemys scripta elegans*) | County Line BBQ | 05/06/2024 | 1513791 | 4,713 | 2,588 |
| C11_42P | Plastron | Male | Red-eared Slider (*Trachemys scripta elegans*) | County Line BBQ | 05/06/2024 | 847797 | 5,548 | 2,478 |
| C12_42S | Skin | Male | Red-eared Slider (*Trachemys scripta elegans*) | County Line BBQ | 05/06/2024 | 434447 | 2,399 | 917 |
| C2_18O | Oral | Female | Red-eared Slider (*Trachemys scripta elegans*) | Butler Metro Park | 03/29/2024 | 543391 | 3,981 | 1,923 |
| C2_22P | Plastron | Male | Red-eared Slider (*Trachemys scripta elegans*) | Butler Metro Park | 03/29/2024 | 670989 | 4,002 | 2,260 |
| C3_19P | Plastron | Male | Red-eared Slider (*Trachemys scripta elegans*) | Butler Metro Park | 03/29/2024 | 346026 | 2,657 | 969 |
| C3_4P | Plastron | Female | Red-eared Slider (*Trachemys scripta elegans*) | Butler Metro Park | 03/29/2024 | 843852 | 2,235 | 1,319 |
| C4_25S | Skin | Male | Red-eared Slider (*Trachemys scripta elegans*) | Butler Metro Park | 03/29/2024 | 858952 | 5,631 | 2,752 |
| C4_5S | Skin | Female | Red-eared Slider (*Trachemys scripta elegans*) | UT Pond | 10/11/2023 | 934170 | 6,038 | 2,749 |
| C5_34C | Carapace | Male | Red-eared Slider (*Trachemys scripta elegans*) | Central Market | 04/11/2024 | 702660 | 8,134 | 3,472 |
| C5_9P | Plastron | Female | Red-eared Slider (*Trachemys scripta elegans*) | UT Pond | 10/11/2023 | 559964 | 1,555 | 845 |
| C6_26P | Plastron | Female | Red-eared Slider (*Trachemys scripta elegans*) | Central Market | 04/11/2024 | 485163 | 6,768 | 3,692 |
| C6_34CO | Cloaca | Male | Red-eared Slider (*Trachemys scripta elegans*) | Central Market | 04/11/2024 | 567483 | 1,966 | 965 |
| C7_33P | Plastron | Male | Red-eared Slider (*Trachemys scripta elegans*) | Central Market | 04/11/2024 | 1491360 | 8,689 | 3,936 |
| C8_31S | Skin | Male | Red-eared Slider (*Trachemys scripta elegans*) | Central Market | 04/11/2024 | 776603 | 3,305 | 1,363 |
| C9_41C | Carapace | Male | Texas Cooter (*Pseudemys texana*) | County Line BBQ | 05/06/2024 | 622522 | 13,140 | 4,531 |
| D1_1C | Carapace | Female | Red-eared Slider (*Trachemys scripta elegans*) | UT Pond | 09/16/2023 | 824108 | 4,443 | 2,672 |
| D1_21C | Carapace | Female | Red-eared Slider (*Trachemys scripta elegans*) | Butler Metro Park | 03/29/2024 | 290772 | 3,357 | 1,832 |
| D10_38O | Oral | Male | Red-eared Slider (*Trachemys scripta elegans*) | County Line BBQ | 05/06/2024 | 1347453 | 2,415 | 1,323 |
| D11_43P | Plastron | Male | Red-eared Slider (*Trachemys scripta elegans*) | County Line BBQ | 05/06/2024 | 858615 | 3,548 | 1,527 |
| D12_44S | Skin | Male | Red-eared Slider (*Trachemys scripta elegans*) | County Line BBQ | 05/06/2024 | 547594 | 3,515 | 1,306 |
| D2_19O | Oral | Male | Red-eared Slider (*Trachemys scripta elegans*) | Butler Metro Park | 03/29/2024 | 1271413 | 4,363 | 2,671 |
| D2_4CO | Cloaca | Female | Red-eared Slider (*Trachemys scripta elegans*) | UT Pond | 10/11/2023 | 1431805 | 6,247 | 3,755 |
| D3_21P | Plastron | Female | Red-eared Slider (*Trachemys scripta elegans*) | Butler Metro Park | 03/29/2024 | 767263 | 1,671 | 962 |
| D3_7P | Plastron | Female | Red-eared Slider (*Trachemys scripta elegans*) | UT Pond | 10/11/2023 | 588535 | 2,000 | 1,120 |
| D4_26C | Carapace | Female | Red-eared Slider (*Trachemys scripta elegans*) | Butler Metro Park | 03/29/2024 | 669352 | 8,341 | 3,180 |
| D4_7S | Skin | Female | Red-eared Slider (*Trachemys scripta elegans*) | UT Pond | 10/11/2023 | 1217498 | 5,669 | 3,698 |
| D5_35C | Carapace | Female | Red-eared Slider (*Trachemys scripta elegans)* | Central Market | 04/11/2024 | 608449 | 6,562 | 2,856 |
| D5_4O | Oral | Female | Red-eared Slider (*Trachemys scripta elegans*) | UT Pond | 10/11/2023 | 444112 | 2,801 | 1,041 |
| D6_28O | Oral | Male | Red-eared Slider (*Trachemys scripta elegans*) | Central Market | 04/11/2024 | 1279457 | 7,948 | 4,278 |
| D6_33O | Oral | Male | Red-eared Slider (*Trachemys scripta elegans*) | Central Market | 04/11/2024 | 1309946 | 9,294 | 5,141 |
| D7_34P | Plastron | Male | Red-eared Slider (*Trachemys scripta elegans*) | Central Market | 04/11/2024 | 705130 | 6,836 | 3,129 |
| D8_32S | Skin | Male | Red-eared Slider (*Trachemys scripta elegans*) | Central Market | 04/11/2024 | 278386 | 2,604 | 605 |
| D9_42C | Carapace | Male | Red-eared Slider (*Trachemys scripta elegans*) | County Line BBQ | 05/06/2024 | 848101 | 7,396 | 2,665 |
| E1_22C | Carapace | Male | Red-eared Slider (*Trachemys scripta elegans*) | Butler Metro Park | 03/29/2024 | 439843 | 3,109 | 1,557 |
| E1_2C | Carapace | Male | Red-eared Slider (*Trachemys scripta elegans*) | UT Pond | 10/11/2023 | 653909 | 4,069 | 2,343 |
| E10_40O | Oral | Male | Red-eared Slider (*Trachemys scripta elegans*) | County Line BBQ | 05/06/2024 | 1905520 | 7,187 | 3,990 |
| E11_44P | Plastron | Male | Red-eared Slider (*Trachemys scripta elegans*) | County Line BBQ | 05/06/2024 | 646258 | 3,416 | 1,361 |
| E12_36C | Carapace | Female | Red-eared Slider (*Trachemys scripta elegans*) | Lake Mueller | 04/19/2024 | 631026 | 8,523 | 3,023 |
| E2_1O | Oral | Female | Red-eared Slider (*Trachemys scripta elegans*) | UT Pond | 09/16/2023 | 1059104 | 2,818 | 1,895 |
| E2_24O | Oral | Male | Red-eared Slider (*Trachemys scripta elegans*) | Butler Metro Park | 03/29/2024 | 576753 | 2,415 | 1,121 |
| E3_23P | Plastron | Female | Red-eared Slider (*Trachemys scripta elegans*) | Butler Metro Park | 03/29/2024 | 657412 | 3,682 | 1,774 |
| E3_8P | Plastron | Female | Red-eared Slider (*Trachemys scripta elegans*) | UT Pond | 10/11/2023 | 738234 | 3,005 | 1,647 |
| E4_28C | Carapace | Male | Red-eared Slider (*Trachemys scripta elegans*) | Central Market | 04/11/2024 | 882694 | 9,998 | 4,655 |
| E4_8S | Skin | Female | Red-eared Slider (*Trachemys scripta elegans*) | UT Pond | 10/11/2023 | 563028 | 2,758 | 1,343 |
| E5_16CO | Cloaca | Male | Red-eared Slider (*Trachemys scripta elegans)* | Butler Metro Park | 03/29/2024 | 436251 | 1,348 | 606 |
| E5_26CO | Cloaca | Female | Red-eared Slider (*Trachemys scripta elegans*) | Central Market | 04/11/2024 | 1415378 | 6,947 | 3,848 |
| E6_14P | Plastron | Female | Red-eared Slider (*Trachemys scripta elegans*) | UT Pond | 11/1/2023 | 390615 | 2,393 | 1,520 |
| E6_29O | Oral | Female | Red-eared Slider (*Trachemys scripta elegans*) | Central Market | 04/11/2024 | 1259505 | 6,376 | 3,613 |
| E7_35P | Plastron | Female | Red-eared Slider (*Trachemys scripta elegans*) | Central Market | 04/11/2024 | 553897 | 4,859 | 2,324 |
| E8_33S | Skin | Male | Red-eared Slider (*Trachemys scripta elegans*) | Central Market | 04/11/2024 | 882630 | 5,865 | 2,293 |
| E9_43C | Carapace | Male | Red-eared Slider (*Trachemys scripta elegans*) | County Line BBQ | 05/06/2024 | 947601 | 14,788 | 5,224 |
| F1_23C | Carapace | Female | Red-eared Slider (*Trachemys scripta elegans*) | Butler Metro Park | 03/29/2024 | 645537 | 4,773 | 1,917 |
| F1_3C | Carapace | Female | Red-eared Slider (*Trachemys scripta elegans*) | UT Pond | 10/11/2023 | 521745 | 2,651 | 1,208 |
| F10_41O | Oral | Male | Texas Cooter (*Pseudemys texana*) | County Line BBQ | 05/06/2024 | 1033475 | 2,746 | 1,616 |
| F11_43S | Skin | Male | Red-eared Slider (*Trachemys scripta elegans*) | County Line BBQ | 05/06/2024 | 818497 | 4,872 | 2,168 |
| F12_37C | Carapace | Female | Red-eared Slider (*Trachemys scripta elegans*) | Lake Mueller | 04/19/2024 | 530315 | 6,287 | 2,246 |
| F2_25O | Oral | Male | Red-eared Slider (*Trachemys scripta elegans*) | Butler Metro Park | 03/29/2024 | 1220265 | 3,061 | 1,872 |
| F2_2O | Oral | Male | Texas Cooter (*Pseudemys texana*) | UT Pond | 10/11/2023 | 1532026 | 7,166 | 4,136 |
| F3_13P | Plastron | Male | Red-eared Slider (*Trachemys scripta elegans*) | UT Pond | 11/1/2023 | 579919 | 4,772 | 2,824 |
| F3_24P | Plastron | Male | Red-eared Slider (*Trachemys scripta elegans*) | Butler Metro Park | 03/29/2024 | 962796 | 2,660 | 1,549 |
| F4_29C | Carapace | Female | Red-eared Slider (*Trachemys scripta elegans*) | Central Market | 04/11/2024 | 580037 | 8,043 | 3,333 |
| F4_9S | Skin | Female | Red-eared Slider (*Trachemys scripta elegans*) | UT Pond | 10/11/2023 | 796186 | 3,641 | 1,914 |
| F5_10P | Plastron | Female | Red-eared Slider (*Trachemys scripta elegans*) | UT Pond | 10/11/2023 | 540111 | 4,025 | 2,335 |
| F5_27CO | Cloaca | Male | Red-eared Slider (*Trachemys scripta elegans*) | Central Market | 04/11/2024 | 463881 | 3,045 | 1,152 |
| F6_30CO | Cloaca | Female | Red-eared Slider (*Trachemys scripta elegans*) | Central Market | 04/11/2024 | 1391476 | 9,537 | 5,187 |
| F6_34O | Oral | Male | Red-eared Slider (*Trachemys scripta elegans*) | Central Market | 04/11/2024 | 1217677 | 5,370 | 3,058 |
| F7_26S | Skin | Female | Red-eared Slider (*Trachemys scripta elegans*) | Central Market | 04/11/2024 | 673668 | 4,432 | 1,728 |
| F8_34S | Skin | Male | Red-eared Slider (*Trachemys scripta elegans*) | Central Market | 04/11/2024 | 633623 | 4,205 | 1,906 |
| F9_44C | Carapace | Male | Red-eared Slider (*Trachemys scripta elegans*) | County Line BBQ | 05/06/2024 | 895231 | 12,471 | 4,816 |
| G1_17S | Skin | Male | Red-eared Slider (*Trachemys scripta elegans*) | Butler Metro Park | 03/29/2024 | 493482 | 2,672 | 1,088 |
| G10_38P | Plastron | Male | Red-eared Slider (*Trachemys scripta elegans*) | County Line BBQ | 05/06/2024 | 450444 | 3,200 | 1,503 |
| G11_47P | Plastron | Female | Red-eared Slider (*Trachemys scripta elegans*) | County Line BBQ | 05/06/2024 | 1647788 | 15,680 | 7,264 |
| G12_37CO | Cloaca | Female | Red-eared Slider (*Trachemys scripta elegans*) | Lake Mueller | 04/19/2024 | 432780 | 1,554 | 687 |
| G2_10O | Oral | Female | Red-eared Slider (*Trachemys scripta elegans*) | UT Pond | 10/11/2023 | 956744 | 2,756 | 1,747 |
| G2_2OP | Plastron | Male | Red-eared Slider (*Trachemys scripta elegans*) | Butler Metro Park | 03/29/2024 | 590899 | 3,419 | 1,485 |
| G3_14P | Plastron | Female | Red-eared Slider (*Trachemys scripta elegans*) | UT Pond | 11/1/2023 | 1067951 | 5,098 | 2,668 |
| G3_25P | Plastron | Male | Red-eared Slider (*Trachemys scripta elegans*) | Butler Metro Park | 03/29/2024 | 749282 | 4,996 | 2,227 |
| G4_11S | Skin | Female | Red-eared Slider (*Trachemys scripta elegans*) | UT Pond | 11/1/2023 | 1134438 | 3,589 | 2,544 |
| G4_30C | Carapace | Female | Red-eared Slider (*Trachemys scripta elegans*) | Central Market | 04/11/2024 | 587725 | 5,522 | 2,397 |
| G5_18C | Carapace | Female | Red-eared Slider (*Trachemys scripta elegans*) | Butler Metro Park | 03/29/2024 | 331105 | 3,088 | 1,443 |
| G5_23O | Oral | Female | Red-eared Slider (*Trachemys scripta elegans*) | Butler Metro Park | 03/29/2024 | 1353592 | 6,988 | 3,249 |
| G6_13S | Skin | Male | Red-eared Slider (*Trachemys scripta elegans*) | UT Pond | 11/1/2023 | 4133299 | 1,853 | 1,012 |
| G6_28P | Plastron | Male | Red-eared Slider (*Trachemys scripta elegans*) | Central Market | 04/11/2024 | 1346794 | 10,220 | 4,878 |
| G7_27S | Skin | Male | Red-eared Slider (*Trachemys scripta elegans*) | Central Market | 04/11/2024 | 1183022 | 9,552 | 4,380 |
| G8_35S | Skin | Female | Red-eared Slider (*Trachemys scripta elegans*) | Central Market | 04/11/2024 | 852870 | 3,992 | 1,990 |
| G9_45C | Carapace | Male | Red-eared Slider (*Trachemys scripta elegans*) | County Line BBQ | 05/06/2024 | 1221369 | 12,385 | 5,546 |
| H1_25C | Carapace | Male | Red-eared Slider (*Trachemys scripta elegans*) | Butler Metro Park | 03/29/2024 | 306837 | 5,924 | 2,726 |
| H1_7C | Carapace | Female | Red-eared Slider (*Trachemys scripta elegans*) | UT Pond | 10/11/2023 | 379622 | 3,608 | 1,966 |
| H10_39P | Plastron | Male | Red-eared Slider (*Trachemys scripta elegans*) | County Line BBQ | 05/06/2024 | 254086 | 1,764 | 802 |
| H11_38S | Skin | Male | Red-eared Slider (*Trachemys scripta elegans*) | County Line BBQ | 05/06/2024 | 29755 | 452 | 116 |
| H12_36P | Plastron | Female | Red-eared Slider (*Trachemys scripta elegans)* | Lake Mueller | 04/19/2024 | 252497 | 1,440 | 636 |
| H2_11O | Oral | Female | Red-eared Slider (*Trachemys scripta elegans*) | UT Pond | 11/1/2023 | 1217448 | 5,042 | 3,007 |
| H2_2OS | Skin | Male | Red-eared Slider (*Trachemys scripta elegans*) | Butler Metro Park | 03/29/2024 | 524057 | 2,921 | 1,428 |
| H3_1S | Skin | Female | Red-eared Slider (*Trachemys scripta elegans*) | UT Pond | 09/16/2023 | 712861 | 2,567 | 1,607 |
| H3_21S | Skin | Female | Red-eared Slider (*Trachemys scripta elegans*) | Butler Metro Park | 03/29/2024 | 573301 | 2,469 | 1,210 |
| H4_13S | Skin | Male | Red-eared Slider (*Trachemys scripta elegans*) | Butler Metro Park | 03/29/2024 | 1384578 | 6,217 | 3,858 |
| H4_31C | Carapace | Male | Red-eared Slider (*Trachemys scripta elegans*) | Central Market | 04/11/2024 | 197661 | 4,250 | 1,723 |
| H5_1P | Plastron | Female | Red-eared Slider (*Trachemys scripta elegans*) | UT Pond | 09/16/2023 | 1012644 | 4,292 | 2,368 |
| H5_29CO | Cloaca | Female | Red-eared Slider (*Trachemys scripta elegans*) | Central Market | 04/11/2024 | 293226 | 2,111 | 686 |
| H6_25CO | Cloaca | Male | Red-eared Slider (*Trachemys scripta elegans*) | Butler Metro Park | 03/29/2024 | 1809221 | 6,952 | 4,078 |
| H6_29P | Plastron | Female | Red-eared Slider (*Trachemys scripta elegans*) | Central Market | 04/11/2024 | 755683 | 4,675 | 2,239 |
| H7_28S | Skin | Male | Red-eared Slider (*Trachemys scripta elegans*) | Central Market | 04/11/2024 | 430829 | 3,864 | 1,605 |
| H8_38C | Carapace | Male | Red-eared Slider (*Trachemys scripta elegans*) | County Line BBQ | 05/06/2024 | 401698 | 7,660 | 2,909 |
| H9_20C | Carapace | Male | Red-eared Slider (*Trachemys scripta elegans*) | Butler Metro Park | 03/29/2024 | 356796 | 2,988 | 1,088 |


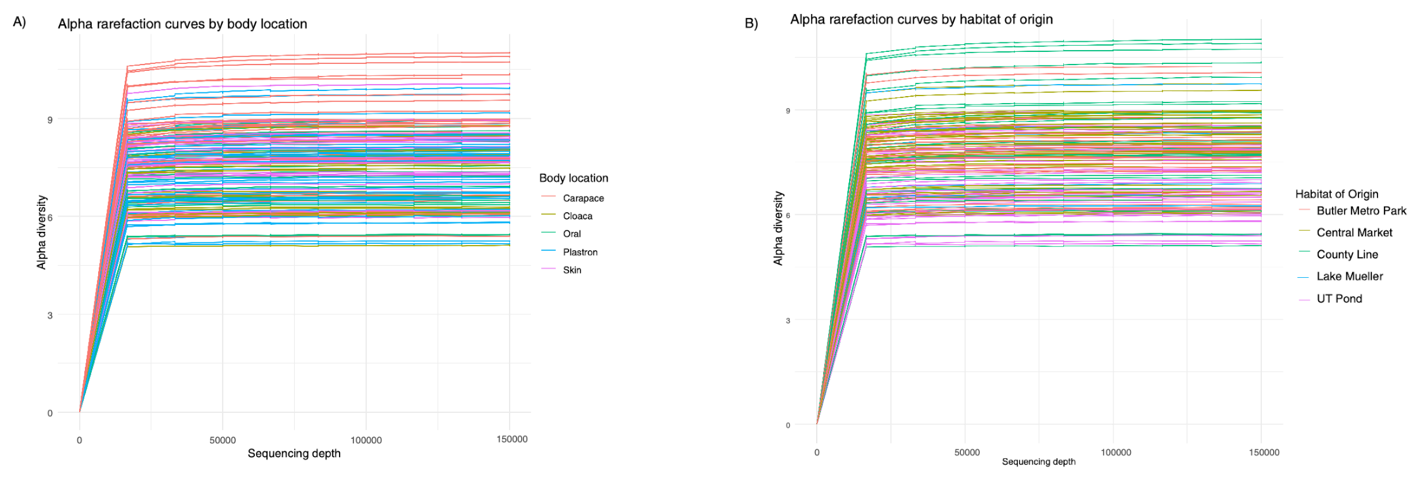


Figure S1. (A) Alpha rarefaction curves alpha diversity (y-axis) and sequencing depth (x-axis) Each line represents an individual sample, with color indicating body location. (B) Alpha rarefaction curves alpha diversity (y-axis) and sequencing depth (x-axis) Each line represents an individual sample, with color indicating Habitat of origin.


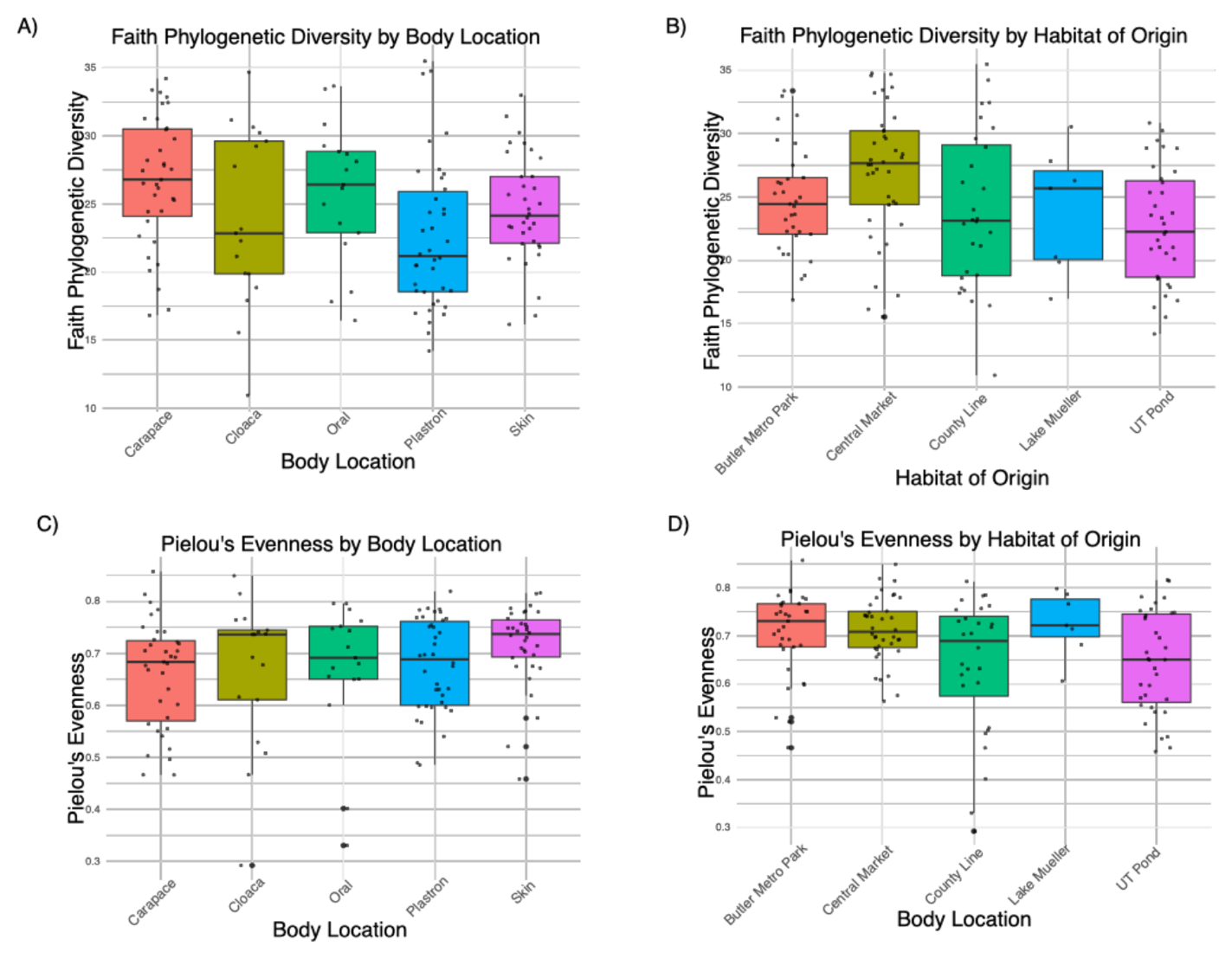


Figure S2. A) Faith Phylogenetic Diversity index across body locations. B) Faith Phylogenetic Diversity index across collection sites. C) Pielou’s evenness index across body locations. D) Pielou’s evenness index across habitat of origin.


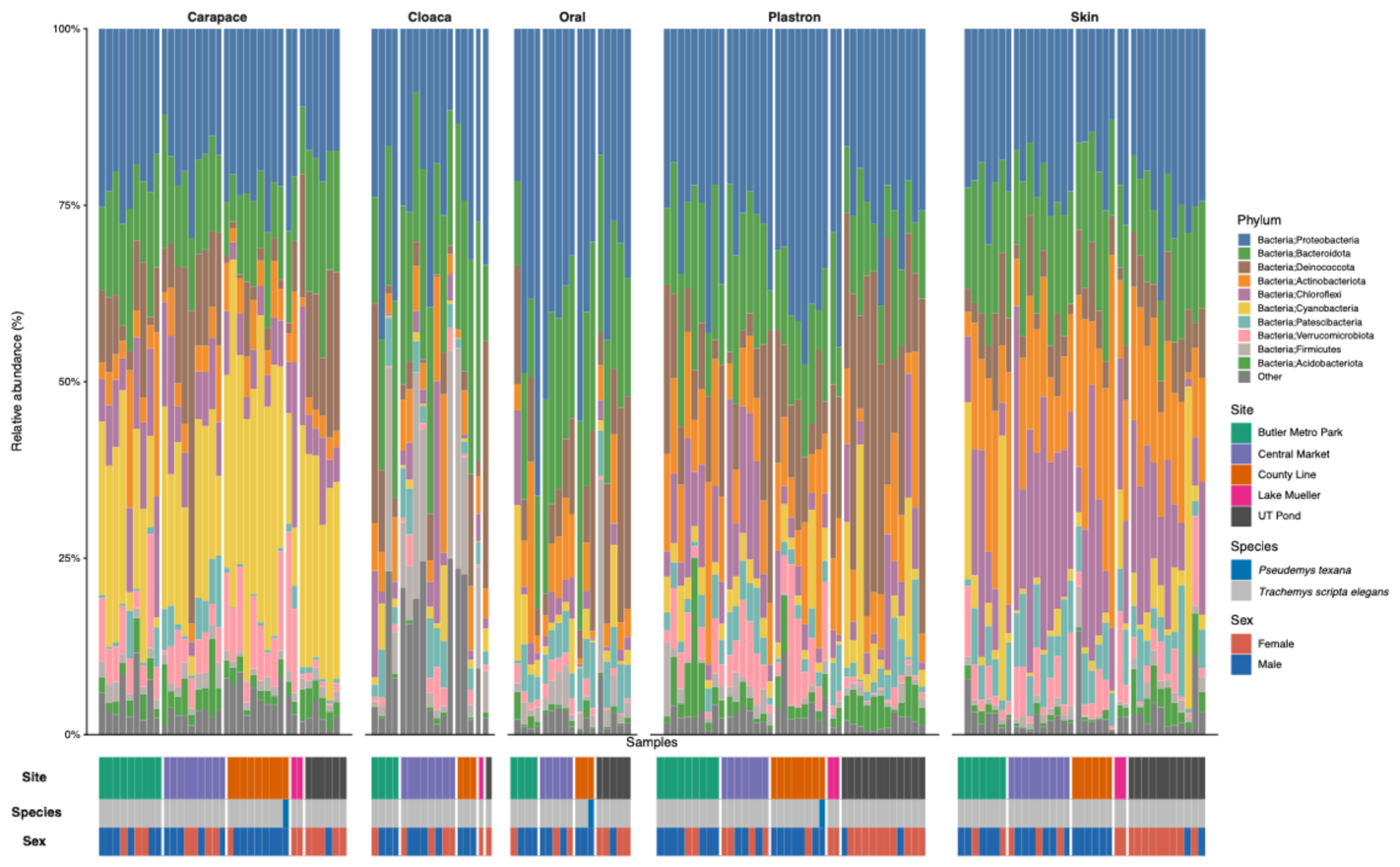


Figure S3. Unfiltered Microbiome 16S rRNA profiles across body sites and habitats from red-eared sliders based on the V3-V4 region. Samples are grouped according to body location, which explains the most variation in microbial diversity, and then by habitat of origin.


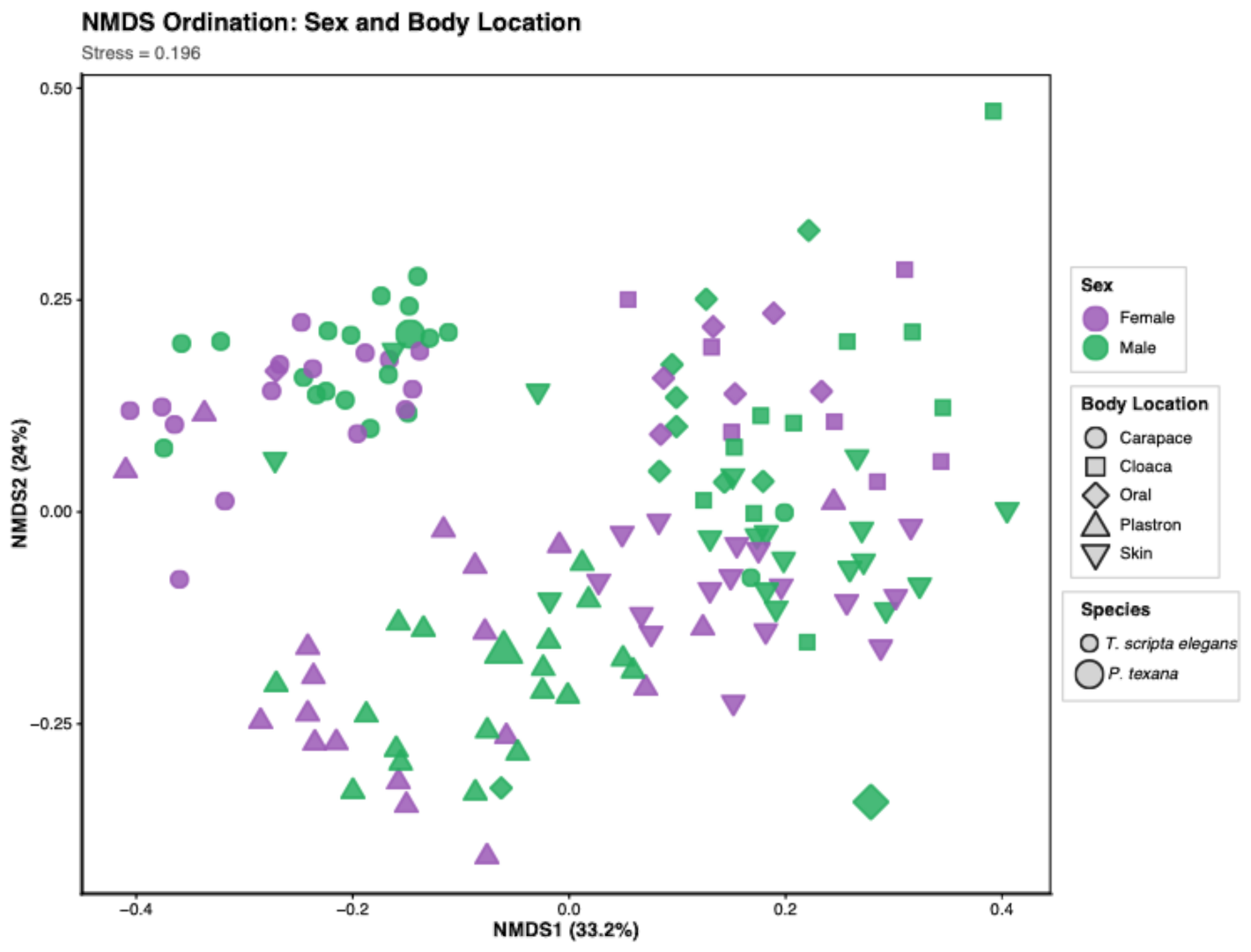


Figure S4. Non-metric multidimensional scaling (NMDS) ordination of Bray Curtis diversity, visualizing dissimilarities in microbial communities across location and sex (stress = 0.196), based on body location and sex.


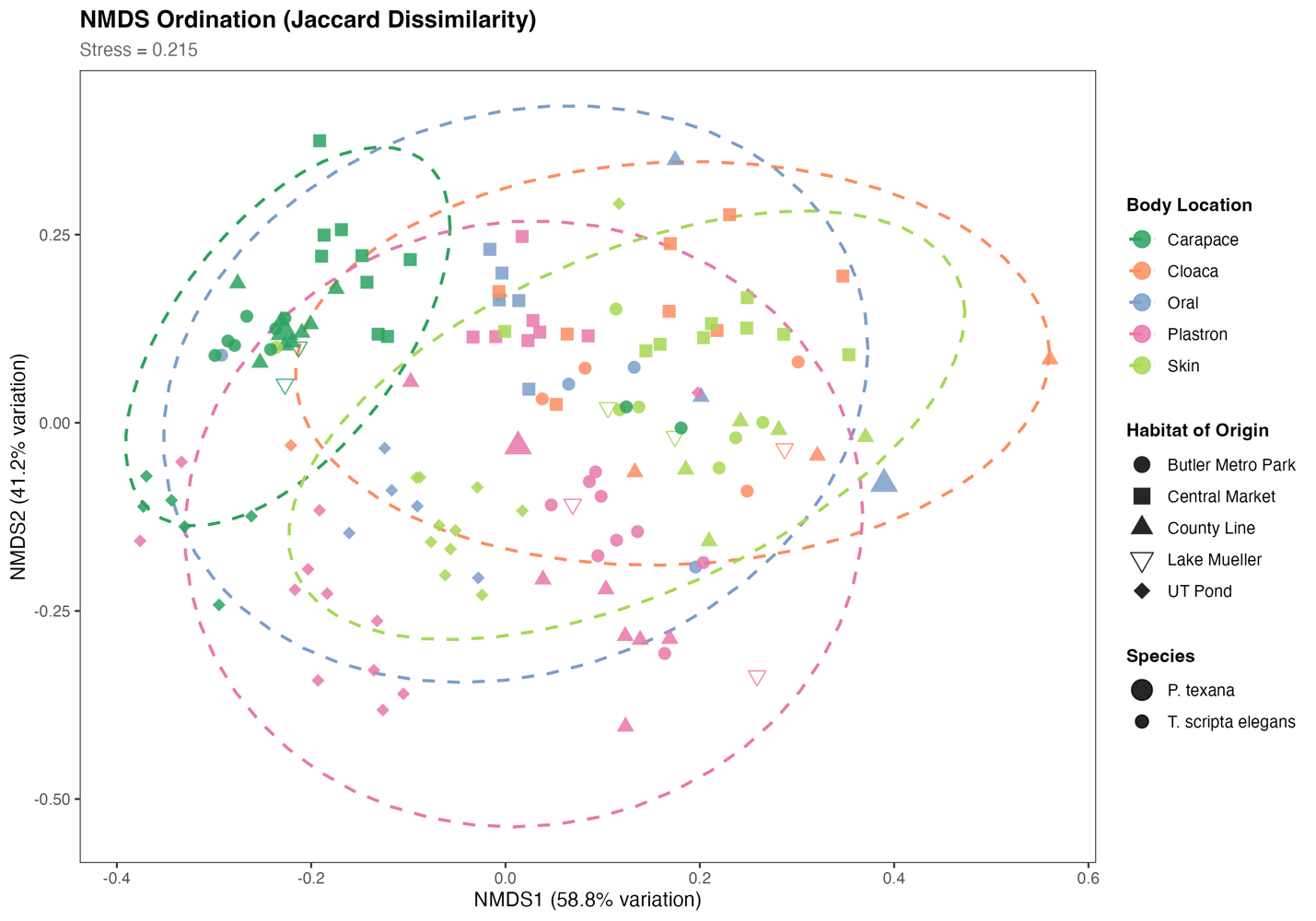


Figure S5. Non-metric multidimensional scaling (NMDS) ordinations of Binary Jaccard beta diversity, visualizing dissimilarities in microbial communities across samples (stress = 0.215). Samples are colored by body location and shapes represent habitat of origin.
