# Supplementary material for: Comparative analysis of the mucosal and shell microbiota of Trachemys scripta elegans across multiple urban freshwater habitats
Source: PLoS One. 2026 Jul 10;21(7):e0353172. doi: 10.1371/journal.pone.0353172 (PMC13353938; doi:10.1371/journal.pone.0353172)
Supplement: Attachment — Submitted filename: R2R_26-01884_Turtle_paper_MCO_JCH_060126.docx [file pone.0353172.s003.docx]

In addition to the reviewer comments addressed below, we have also resolved the following editorial requests from the PLOS ONE editorial office:

- **Figure 1 caption** has been updated to include the data source for map shapefiles (U.S. Census Bureau TIGER/Line, public domain via R tigris package).
- **Figures have been removed** from the manuscript file and submitted as separate TIFF files.
- **Funder role statement** has been added.

We thank the reviewers for their insightful comments, which have greatly improved our manuscript. Below, we provide a point-by-point response to these comments, our efforts to incorporate them into the current manuscript, and, at times, our reasoning for not incorporating them (line numbers refer to tracked changes of version).

Referee: 1
This manuscript presents a timely and well-structured investigation into the microbial communities associated with an invasive freshwater turtle, the red-eared slider, across urban habitats in Austin, Texas. The study employs 16S rRNA amplicon sequencing to characterize microbial profiles from five body sites and compares them across several urban ponds/lakes. The topic is relevant to microbial ecology, invasion of biology, and public health, and the inclusion of student researchers adds an educational dimension to the work. While the core findings are clear and well-supported by the data, several methodological and interpretational aspects could be strengthened to enhance the manuscript's impact and clarity. But there are still some major concerns and suggestions:

We thank reviewer 1 for their positive comments and we incorporated their suggestions to improve our study.

1.The introduction effectively reviews sea turtle microbiomes but could briefly reference key freshwater turtle microbiome studies beyond the red-eared slider to better contextualize the work.

Agreed. We have added lines on freshwater turtle microbiome studies beyond red-eared sliders (line 80-87)

2.The absence of water/sediment samples from each habitat limits the ability to distinguish between environmentally derived vs. host-specific microbes. In Discussion Section P303-305, the higher relative abundance of Cyanobacteria is relative to the environment. Additionally, future work should integrate paired host–environment sampling across habitats to disentangle these influences rigorously. Moreover, the temporal dimension is also overlooked.

We agree that the absence of paired environmental samples limits our ability to conclusively distinguish environmentally derived from host-specific microbes. In the revised Discussion (lines 315–317). We have explicitly acknowledged this limitation and clarified that our interpretations regarding the relative abundance of Cyanobacteria are made with caution due to the lack of direct environmental comparisons. We also point out that cloacal/oral samples are more likely to be indicative of gut microbiota, while those on the shell/skin are more likely influenced by the environment. We also point out that future studies should incorporate environmental sampling. Finally, we also now suggest that future studies should examine microbiomes across time/seasons to determine how temporal effects change microbiomes in different body regions (lines 403-409).

3. Sample size imbalance. Only two turtles were sampled from Lake Muller, compared to 10–14 from other sites. This may affect habitat-level comparisons and statistical power. Please justify this disparity or acknowledge it as a study limitation.

Fewer turtles were sampled from Lake Mueller because it was a larger body of water, making them harder to catch than at other sites. Rather than remove these samples from the analyses, we now have addressed these technical constraints in the methodology section, lines 155-156, acknowledging that a limited sample size from this habitat makes inferences about its population limited (lines 414-415).

4.The 16S rRNA approach provides community profiling but limits functional interpretation. A brief discussion on the potential metabolic roles of dominant taxa (e.g., Cyanobacteria on shells) would enrich the ecological narrative. If possible, mention whether any PICRUSt2 or similar inference was attempted, even if only preliminarily.

We thank the reviewer for the suggestion on using PiCRUSt2 for looking into metabolic roles of dominant taxa. In response, we did run PICRUSt. We found that potential metabolic profiles of the samples did vary based on body habitat, supporting our ASV diversity results (figure below). However, functional differences in microbiota were not the focus on this project. As our identification was only based on a small region of the 16S rRNA, we do not fully trust the metabolic inferences and prefer to not make them a focus of the current study. Therefore, we suggest a potential future project could use whole genome sequencing to look more convincingly at different metabolic profiles and functional diversity of turtle microbiomes.

5. The results are too simple. Some findings in discussion are not explained in the Result Sections.

We’re not sure what findings/results the reviewer is referring to but have addressed similar comments from the other reviewer that we hope address this comment. We do agree that our results are relatively simple, but do not see this as a limitation. We have made sure that the Results and the Discussion sections are more synced.

6. Pathogen discussion could be contextualized. While potential pathogens (e.g., Acinetobacter, Mycobacterium) are noted, their low abundance is appropriately highlighted. However, the public health implications could be better framed by referencing local or national guidelines on zoonotic risk from handled turtles.

We have softened the claims and added line 361-366 on future research of pathogen screening and added context on the zoonotic risk of handling turtles.

7. The discussion on extremophiles (e.g., Chloroflexi, Halobacteriota) is interesting but somewhat speculative. Consider toning down claims about “behavior facilitating colonization” unless direct evidence or citations are provided.

We have removed the claims about behavior facilitating colonization and the excess discussion on extremophiles.

Referee 2:

This manuscript investigates the microbiome associated with different body regions of the invasive red-eared slider turtle (Trachemys scripta elegans) across several urban freshwater habitats using 16S rRNA sequencing. The study addresses an interesting and relevant topic in microbial ecology, invasive species biology, and urban ecosystem research. The dataset includes multiple body locations (carapace, plastron, skin, oral cavity, cloaca) and several sampling sites, which are valuable for understanding microbiome structure in reptiles.

However, the manuscript requires substantial revision before it can be considered for publication.

Several methodological aspects are insufficiently explained; statistical analyses need clarification, and parts of the discussion overinterpret the results. In addition, the manuscript contains inconsistencies in sampling description, analytical procedures, and interpretation of microbiome data.

Overall, the study has potential, but major revisions are necessary to improve methodological transparency, strengthen statistical analyses, and better align conclusions with the presented data.

We thank the reviewer for their useful comments and the manuscript improved by them.

The abstract states that: “community composition strongly structured by body location rather than habitat.” However, PERMANOVA results show that both factors were significant. This should be clarified.

We thank the reviewer for this observation. We have revised the abstract to clarify that both body location and habitat of origin significantly influenced microbial community composition.

The description of sampling sites and sampling dates is inconsistent and confusing. For example, in the methods section, sampling is reported to occur between September 2023 and May 2024, yet Lake Muller samples are reported from April 2025 (page ~13). The study refers to five locations, but the site descriptions and sample counts are difficult to follow. A table summarizing the sampling effort should be added. This will greatly improve clarity.

We thank the reviewer for pointing out these inconsistencies, which mainly stemmed from some typos and similar errors when preparing the manuscript. Sampling occurred between September 2023 and May 2024; the reference to April 2025 for the Lake Muller samples was a typo, which has been corrected on line 155. To further improve clarity, we have added a table (Table S1) summarizing the sampling effort in the supplemental information to strengthen clarity for the readers.

The study includes two individuals of Pseudemys texana among the samples (Fig. 2 captions). This raises several issues: Why were individuals from different species included in the microbiome analysis? Were they included in statistical analysis? If so, how was species identity controlled? Because microbiomes are often host-species specific, mixing species may bias the results. The authors should either: remove these samples from the analysis or clearly justify their inclusion and statistically control for species effects.

Individuals of *Pseudemys texana* were collected opportunistically. Given the lack of prior microbiome data for this species, we included these samples to provide preliminary reference data and contribute to publicly available datasets.

We agree that differences in host species can influence microbiome composition. To account for this, species identity was included as a factor in the PERMANOVA models. Species did not explain a significant portion of variation in microbial community composition (R² = 0.003, p = 0.920), indicating that inclusion of these samples did not bias the overall results, although we acknowledge a more focused study with larger sample sizes for *P. texana* would be needed to appropriately test this.

We acknowledge that the comparison is highly unbalanced (n = 139 vs. n = 3 samples) and therefore underpowered to detect species-level effects. We have clarified this limitation in the revised manuscript and justified including these samples to aid future work (lines 317-318). We have also changed the figures to make it clear which samples are from *P. texana*.

The authors state that rarefaction depth was set to 600,000 reads (Methods section). However: The average read count per sample is ~779,776 reads. Rarefaction at such a high depth may remove a large proportion of samples. The manuscript must clarify: How many samples were retained after rarefaction, whether rarefaction curves justified this threshold, and whether lower rarefaction depths were tested.

We thank the reviewer for pointing out this inconsistency. The originally reported rarefaction depth of 600,000 reads was incorrect and has been revised in the manuscript. Following DADA2 quality filtering and denoising, the number of usable reads per sample was substantially reduced relative to the initial sequencing depth. Alpha rarefaction analyses demonstrated that diversity plateaued between approximately 100,000–150,000 reads (Fig. S2), indicating that sequencing depth was sufficient to capture most microbial diversity across samples. Based on these results, analyses were conducted using a rarefaction depth of 150,000 reads. We have clarified this in the revised Methods section and updated the manuscript accordingly (Lines 215-216).

The authors filtered ASVs to those: “present in at least 50 of 142 samples.” This threshold is extremely strict and will eliminate rare taxa, which can be ecologically important in microbiome studies. The authors should: justify this filtering strategy: evaluate whether it removed site- or body-specific taxa, report the number of ASVs before and after filtering.

We agree that the threshold was overly strict, so we changed the filtering criterion to ASVs present in at least 5 of 142 samples. This increased the number of retained ASVs, and the numbers before and after filtering are now included in Supplemental Table 2. Importantly, this change did not substantially alter the overall results or interpretations of the study.

The manuscript reports that sex did not explain variation, yet the PERMANOVA result shows: F = 3.28, p = 0.001. A p-value of 0.001 indicates significance, contradicting the interpretation. This discrepancy must be corrected. PERMANOVA assumes homogeneous dispersion among groups. The manuscript does not report whether PERMDISP tests were conducted. Without this test, the interpretation of PERMANOVA results is incomplete. The NMDS stress value is 0.196. This value is relatively high and indicates a moderate representation of the data.

The originally reported PERMANOVA result for sex (p = 0.001) was based on a univariate model. We have now reanalyzed the data using a multivariable PERMANOVA including site and body location, which revealed that sex does not explain a significant portion of variation after accounting for these variables (PERMANOVA, R² = 0.006, p = 0.142). This indicates that the previously observed effect of sex was because it was tested on its own and not taking into consideration body location and habitat of origin. We have updated the manuscript accordingly.

In addition, based on the reviewer’s comment we conducted PERMDISP analyses to assess homogeneity of dispersion among groups. No significant differences in dispersion were detected for body location, habitat, or sex (all p > 0.05), confirming that PERMANOVA results reflect true compositional differences rather than differences in group variance. These results are now reported in the revised manuscript (lines 310-313).

The authors should: report whether additional dimensions were tested and discuss limitations of the ordination. The discussion repeatedly suggests that turtle microbiomes reflect environmental microbial communities. However: “we did not include environmental water samples.” This severely limits interpretation. Without environmental samples, claims about environmental influence remain speculative. The authors should: explicitly acknowledge this limitation and reduce speculation regarding environmental microbial sources.

We agree and we have included our limitation from not having environmental samples and we have reduced the speculation regarding environmental microbial resources (lines 319-322). Additional ordination dimensions were evaluated using a 3-dimensional NMDS. The 3-dimensional solution reduced stress from 0.196 to 0.141, indicating improved representation of community dissimilarities. However, the 2-dimensional NMDS was retained for visualization purposes and ease of interpretation.

The manuscript identifies genera containing pathogens such as: Acinetobacter, Mycobacterium and Roseomonas, however: 16S rRNA sequencing cannot identify pathogenic strains; many species in these genera are harmless environmental microbes. Therefore, statements suggesting public health risk should be softened unless supported by targeted pathogen screening.

We agreed that many of these genera are harmless environmental microbes, and we have softened the claims and added a line on future research of pathogen screening (lines 392-394). We also note this comment underlies our response to R1 above about how only limited functional inference can be made based on 16S data.

The discussion contains several paragraphs that extend beyond the scope of the results. Examples include extensive speculation about extremophiles, educational programs and student participation, and environmental pollution pathways. These sections dilute the scientific focus of the manuscript. The discussion should be condensed and focused on microbiome findings.

We feel some speculation in the Discussion is warranted to spur future research, but also agree with the reviewer that too much speculation may dilute the scientific merit of the manuscript. We therefore removed the discussion on extremophiles from the manuscript (also in accordance with R1’s comments). However, as a major point of this work was to develop an undergraduate-based research program on biodiversity, we feel it is important to include this perspective in the manuscript. To make this clearer, we have included more information to the introduction of the manuscript to highlight the potential role of urban ponds in student-led research and the motivation behind our work (line 131-134).

Figure 2: The stacked bar plot is very crowded. Consider summarizing class level rather than phylum level.

We are unsure about this comment, as summarizing the stacked bar plot at the class level rather than phylum level would make it even more crowded (i.e., there are multiple classes per phyla, so there would be many more colors). Below is an example of what the bar plot looks like at the phylum (A) vs. class level (B). We have chosen to keep the display at the phyla level (the broadest taxonomic grouping available in QIIME) but are certainly open to other ways to improve the clarity of this figure.

Figure 3: Axis labels should include variance explained if available. Symbols representing habitats are difficult to distinguish. Several bacterial genera appear to be misspelled.

We have altered the symbols representing the habitats to increase clarity. We’re not sure of what bacterial genera the reviewer is referencing here since the figure illustrates the habitat of origin and the body location where the samples were collected with no genera mentioned. However, we have checked genera spellings throughout the manuscript.

The manuscript addresses an interesting topic and presents a potentially valuable dataset. However, significant revisions are required to improve methodological clarity, correct inconsistencies, and strengthen the statistical analyses before the study can be considered for publication.
