# Supplementary material for: Comparative analysis of the mucosal and shell microbiota of Trachemys scripta elegans across multiple urban freshwater habitats
Source: PLoS One. 2026 Jul 10;21(7):e0353172. doi: 10.1371/journal.pone.0353172 (PMC13353938; doi:10.1371/journal.pone.0353172)
Supplement: Peer review history

# Peer review: Decision Letter 0

**DOI:** 10.1371/journal.pone.0353172.r001

**Authors:** Murtada D. Naser

5 Mar 2026

PONE-D-26-01884

Comparative Analysis of the Mucosal and Shell Microbiota of Trachemys scripta elegans Across Multiple Urban Freshwater Habitats

PLOS One

Dear Dr. Correa Orellana,

Thank you for submitting your manuscript to PLOS ONE. After careful consideration, we feel that it has merit but does not fully meet PLOS ONE’s publication criteria as it currently stands. Therefore, we invite you to submit a revised version of the manuscript that addresses the points raised during the review process.

Your manuscript has now been evaluated by two reviewers. Both reviewers find the study interesting and potentially valuable for understanding microbiome variation in invasive freshwater turtles. However, they also identified several important issues that must be addressed before the manuscript can be considered for publication.

In particular, the reviewers raised concerns regarding:

1. clarity and consistency in the sampling design and site descriptions,

2. the inclusion of individuals from another turtle species and its implications for the analyses,

3. the rarefaction depth and ASV filtering strategy, which require further justification and clarification,

4. interpretation of statistical results, including inconsistencies in the reporting of PERMANOVA results,

5. the need to test PERMANOVA assumptions (e.g., homogeneity of dispersion),

6. the interpretation of microbiome–environment relationships in the absence of environmental samples, and

7. discussion points that extend beyond the scope of the presented results.

The reviewers also suggest improving figure clarity, providing a clearer summary of sampling effort, and refining parts of the discussion.

Given these issues, the manuscript requires substantial revision before it can proceed further in the review process.

Please revise the manuscript carefully in light of the reviewers’ comments and provide a detailed point-by-point response explaining how each comment has been addressed.

We look forward to receiving your revised manuscript.

Best regards,

Dr. Murtada D. Naser

Academic Editor

PLOS ONE

Please submit your revised manuscript by Apr 19 2026 11:59PM. If you will need more time than this to complete your revisions, please reply to this message or contact the journal office at plosone@plos.org. When you're ready to submit your revision, log on to https://www.editorialmanager.com/pone/ and select the 'Submissions Needing Revision' folder to locate your manuscript file.

Please include the following items when submitting your revised manuscript:

- A letter that responds to each point raised by the academic editor and reviewer(s). You should upload this letter as a separate file labeled 'Response to Reviewers'.
- A marked-up copy of your manuscript that highlights changes made to the original version. You should upload this as a separate file labeled 'Revised Manuscript with Track Changes'.
- An unmarked version of your revised paper without tracked changes. You should upload this as a separate file labeled 'Manuscript'.

If you would like to make changes to your financial disclosure, please include your updated statement in your cover letter. Guidelines for resubmitting your figure files are available below the reviewer comments at the end of this letter.

If applicable, we recommend that you deposit your laboratory protocols in protocols.io to enhance the reproducibility of your results. Protocols.io assigns your protocol its own identifier (DOI) so that it can be cited independently in the future. For instructions see: https://journals.plos.org/plosone/s/submission-guidelines#loc-laboratory-protocols. Additionally, PLOS ONE offers an option for publishing peer-reviewed Lab Protocol articles, which describe protocols hosted on protocols.io. Read more information on sharing protocols at https://plos.org/protocols?utm_medium=editorial-email&utm_source=authorletters&utm_campaign=protocols.

We look forward to receiving your revised manuscript.

Kind regards,

Murtada D. Naser

Academic Editor

PLOS One

Journal requirements:

When submitting your revision, we need you to address these additional requirements.

1. Please ensure that your manuscript meets PLOS ONE's style requirements, including those for file naming. The PLOS ONE style templates can be found at

https://journals.plos.org/plosone/s/file?id=wjVg/PLOSOne_formatting_sample_main_body.pdf and

https://journals.plos.org/plosone/s/file?id=ba62/PLOSOne_formatting_sample_title_authors_affiliations.pdf

2. In your Methods section, please provide additional information regarding the permits you obtained for the work. Please ensure you have included the full name of the authority that approved the field site access and, if no permits were required, a brief statement explaining why.

3. We note that Figure 1 in your submission contain [map/satellite] images which may be copyrighted. All PLOS content is published under the Creative Commons Attribution License (CC BY 4.0), which means that the manuscript, images, and Supporting Information files will be freely available online, and any third party is permitted to access, download, copy, distribute, and use these materials in any way, even commercially, with proper attribution. For these reasons, we cannot publish previously copyrighted maps or satellite images created using proprietary data, such as Google software (Google Maps, Street View, and Earth). For more information, see our copyright guidelines: http://journals.plos.org/plosone/s/licenses-and-copyright.

We require you to either (1) present written permission from the copyright holder to publish these figures specifically under the CC BY 4.0 license, or (2) remove the figures from your submission:

1. You may seek permission from the original copyright holder of Figure 1 to publish the content specifically under the CC BY 4.0 license.

We recommend that you contact the original copyright holder with the Content Permission Form (http://journals.plos.org/plosone/s/file?id=7c09/content-permission-form.pdf) and the following text:

“I request permission for the open-access journal PLOS ONE to publish XXX under the Creative Commons Attribution License (CCAL) CC BY 4.0 (http://creativecommons.org/licenses/by/4.0/). Please be aware that this license allows unrestricted use and distribution, even commercially, by third parties. Please reply and provide explicit written permission to publish XXX under a CC BY license and complete the attached form.”

Please upload the completed Content Permission Form or other proof of granted permissions as an "Other" file with your submission.

In the figure caption of the copyrighted figure, please include the following text: “Reprinted from [ref] under a CC BY license, with permission from [name of publisher], original copyright [original copyright year].”

2. If you are unable to obtain permission from the original copyright holder to publish these figures under the CC BY 4.0 license or if the copyright holder’s requirements are incompatible with the CC BY 4.0 license, please either i) remove the figure or ii) supply a replacement figure that complies with the CC BY 4.0 license. Please check copyright information on all replacement figures and update the figure caption with source information. If applicable, please specify in the figure caption text when a figure is similar but not identical to the original image and is therefore for illustrative purposes only.

The following resources for replacing copyrighted map figures may be helpful:

USGS National Map Viewer (public domain): http://viewer.nationalmap.gov/viewer/

The Gateway to Astronaut Photography of Earth (public domain): http://eol.jsc.nasa.gov/sseop/clickmap/

Maps at the CIA (public domain): https://www.cia.gov/library/publications/the-world-factbook/index.html and https://www.cia.gov/library/publications/cia-maps-publications/index.html

NASA Earth Observatory (public domain): http://earthobservatory.nasa.gov/

Landsat: http://landsat.visibleearth.nasa.gov/

USGS EROS (Earth Resources Observatory and Science (EROS) Center) (public domain): http://eros.usgs.gov/#

Natural Earth (public domain): http://www.naturalearthdata.com/

4. Please upload a copy of Figures 2 and 3, to which you refer in your text on page 7. If the figure is no longer to be included as part of the submission please remove all reference to it within the text.

5. When completing the data availability statement of the submission form, you indicated that you will make your data available on acceptance. We strongly recommend all authors decide on a data sharing plan before acceptance, as the process can be lengthy and hold up publication timelines. Please note that, though access restrictions are acceptable now, your entire data will need to be made freely accessible if your manuscript is accepted for publication. This policy applies to all data except where public deposition would breach compliance with the protocol approved by your research ethics board. If you are unable to adhere to our open data policy, please kindly revise your statement to explain your reasoning and we will seek the editor's input on an exemption. Please be assured that, once you have provided your new statement, the assessment of your exemption will not hold up the peer review process.

6. Please include captions for your Supporting Information files at the end of your manuscript, and update any in-text citations to match accordingly. Please see our Supporting Information guidelines for more information: http://journals.plos.org/plosone/s/supporting-information.

If the reviewer comments include a recommendation to cite specific previously published works, please review and evaluate these publications to determine whether they are relevant and should be cited. There is no requirement to cite these works unless the editor has indicated otherwise.

Additional Editor Comments:

Dear Authors,

Thank you for submitting your manuscript entitled “Comparative Analysis of the Mucosal and Shell Microbiota of Trachemys scripta elegans Across Multiple Urban Freshwater Habitats” to PLOS ONE.

Your manuscript has now been evaluated by two reviewers. Both reviewers find the study interesting and potentially valuable for understanding microbiome variation in invasive freshwater turtles. However, they also identified several important issues that must be addressed before the manuscript can be considered for publication.

In particular, the reviewers raised concerns regarding:

1. clarity and consistency in the sampling design and site descriptions,

2. the inclusion of individuals from another turtle species and its implications for the analyses,

3. the rarefaction depth and ASV filtering strategy, which require further justification and clarification,

4. interpretation of statistical results, including inconsistencies in the reporting of PERMANOVA results,

5. the need to test PERMANOVA assumptions (e.g., homogeneity of dispersion),

6. the interpretation of microbiome–environment relationships in the absence of environmental samples, and

7. discussion points that extend beyond the scope of the presented results.

The reviewers also suggest improving figure clarity, providing a clearer summary of sampling effort, and refining parts of the discussion.

Given these issues, the manuscript requires substantial revision before it can proceed further in the review process.

Please revise the manuscript carefully in light of the reviewers’ comments and provide a detailed point-by-point response explaining how each comment has been addressed.

We look forward to receiving your revised manuscript.

Best regards,

Dr. Murtada D. Naser

Academic Editor

PLOS ONE

[Note: HTML markup is below. Please do not edit.]

Reviewers' comments:

Reviewer's Responses to Questions

**Comments to the Author**

1. Is the manuscript technically sound, and do the data support the conclusions?

The manuscript must describe a technically sound piece of scientific research with data that supports the conclusions. Experiments must have been conducted rigorously, with appropriate controls, replication, and sample sizes. The conclusions must be drawn appropriately based on the data presented.

Reviewer #1: Yes

Reviewer #2: No

**********

2. Has the statistical analysis been performed appropriately and rigorously?

Reviewer #1: Yes

Reviewer #2: No

**********

3. Have the authors made all data underlying the findings in their manuscript fully available?

The PLOS Data policy requires authors to make all data underlying the findings described in their manuscript fully available without restriction, with rare exception (please refer to the Data Availability Statement in the manuscript PDF file). The data should be provided as part of the manuscript or its supporting information, or deposited to a public repository. For example, in addition to summary statistics, the data points behind means, medians and variance measures should be available. If there are restrictions on publicly sharing data—e.g. participant privacy or use of data from a third party—those must be specified.

Reviewer #1: Yes

Reviewer #2: No

**********

4. Is the manuscript presented in an intelligible fashion and written in standard English?

PLOS ONE does not copyedit accepted manuscripts, so the language in submitted articles must be clear, correct, and unambiguous. Any typographical or grammatical errors should be corrected at revision, so please note any specific errors here.

Reviewer #1: Yes

Reviewer #2: Yes

**********

5. Review Comments to the Author

Please use the space provided to explain your answers to the questions above. You may also include additional comments for the author, including concerns about dual publication, research ethics, or publication ethics. (Please upload your review as an attachment if it exceeds 20,000 characters)

Reviewer #1: This manuscript presents a timely and well-structured investigation into the microbial communities associated with an invasive freshwater turtle, the red-eared slider, across urban habitats in Austin, Texas. The study employs 16S rRNA amplicon sequencing to characterize microbial profiles from five body sites and compares them across several urban ponds/lakes. The topic is relevant to microbial ecology, invasion biology, and public health, and the inclusion of student researchers adds an educational dimension to the work. While the core findings are clear and well-supported by the data, several methodological and interpretational aspects could be strengthened to enhance the manuscript's impact and clarity. But there are still some major concerns and suggestions:

1.The introduction effectively reviews sea turtle microbiomes but could briefly reference key freshwater turtle microbiome studies beyond the red-eared slider to better contextualize the work.

2.The absence of water/sediment samples from each habitat limits the ability to distinguish between environmentally derived vs. host-specific microbes. In Discussion Section P303-305, the higher relative abundance of Cyanobacteria is relative to environment. Additionally, future work should integrate paired host–environment sampling across habitats to disentangle these influences rigorously. Moreover, the temporal dimension is also overlooked.

3.Sample size is imbalance. Only two turtles were sampled from Lake Muller, compared to 10–14 from other sites. This may affect habitat-level comparisons and statistical power. Please justify this disparity or acknowledge it as a study limitation.

4.The 16S rRNA approach provides community profiling but limits functional interpretation. A brief discussion on the potential metabolic roles of dominant taxa (e.g., Cyanobacteria on shells) would enrich the ecological narrative. If possible, mention whether any PICRUSt2 or similar inference was attempted, even if only preliminarily.

5.The results are too simple. Some findings in discussion are not explained in the Result Sections.

6.Pathogen discussion could be contextualized. While potential pathogens (e.g., Acinetobacter, Mycobacterium) are noted, their low abundance is appropriately highlighted. However, the public health implications could be better framed by referencing local or national guidelines on zoonotic risk from handled turtles.

7.The discussion on extremophiles (e.g., Chloroflexi, Halobacteriota) is interesting but somewhat speculative. Consider toning down claims about “behavior facilitating colonization” unless direct evidence or citations are provided.

Reviewer #2: This manuscript investigates the microbiome associated with different body regions of the invasive red-eared slider turtle (Trachemys scripta elegans) across several urban freshwater habitats using 16S rRNA sequencing. The study addresses an interesting and relevant topic in microbial ecology, invasive species biology, and urban ecosystem research. The dataset includes multiple body locations (carapace, plastron, skin, oral cavity, cloaca) and several sampling sites, which is valuable for understanding microbiome structure in reptiles.

However, the manuscript requires substantial revision before it can be considered for publication. Several methodological aspects are insufficiently explained, statistical analyses need clarification, and parts of the discussion overinterpret the results. In addition, the manuscript contains inconsistencies in sampling description, analytical procedures, and interpretation of microbiome data.

Overall, the study has potential, but major revisions are necessary to improve methodological transparency, strengthen statistical analyses, and better align conclusions with the presented data.

The abstract states that: “community composition strongly structured by body location rather than habitat.” However, PERMANOVA results show both factors were significant. This should be clarified. The description of sampling sites and sampling dates is inconsistent and confusing. For example, in the methods section, sampling is reported to occur between September 2023 and May 2024, yet Lake Muller samples are reported from April 2025 (page ~13). The study refers to five locations, but the site descriptions and sample counts are difficult to follow. A table summarizing sampling effort should be added. This will greatly improve clarity. The study includes two individuals of Pseudemys texana among the samples (Fig. 2 caption). This raises several issues: Why were individuals from a different species included in the microbiome analysis? Were they included in statistical analyses? If so, how was species identity controlled? Because microbiomes are often host-species specific, mixing species may bias the results. The authors should either: remove these samples from the analysis or clearly justify their inclusion and statistically control for species effects. The authors state that rarefaction depth was set to 600,000 reads (Methods section). However: The average read count per sample is ~779,776 reads. Rarefaction at such a high depth may remove a large proportion of samples. The manuscript must clarify: How many samples were retained after rarefaction, whether rarefaction curves justified this threshold and whether lower rarefaction depths were tested. The authors filtered ASVs to those: “present in at least 50 of 145 samples.” This threshold is extremely strict and will eliminate rare taxa, which can be ecologically important in microbiome studies. The authors should: justify this filtering strategy: evaluate whether it removed site- or body-specific taxa, report the number of ASVs before and after filtering. The manuscript reports that sex did not explain variation, yet the PERMANOVA result shows: F = 3.28, p = 0.001. A p-value of 0.001 indicates significance, contradicting the interpretation. This discrepancy must be corrected. PERMANOVA assumes homogeneous dispersion among groups. The manuscript does not report whether PERMDISP tests were conducted. Without this test, the interpretation of PERMANOVA results is incomplete. The NMDS stress value is 0.196. This value is relatively high and indicates a moderate representation of the data. The authors should: report whether additional dimensions were tested and discuss limitations of the ordination. The discussion repeatedly suggests that turtle microbiomes reflect environmental microbial communities. However: “we did not include environmental water samples.” This severely limits interpretation. Without environmental samples, claims about environmental influence remain speculative. The authors should: explicitly acknowledge this limitation and reduce speculation regarding environmental microbial sources. The manuscript identifies genera containing pathogens such as: Acinetobacter, Mycobacterium and Roseomonas, however: 16S rRNA sequencing cannot identify pathogenic strains, many species in these genera are harmless environmental microbes. Therefore, statements suggesting public health risk should be softened unless supported by targeted pathogen screening. The discussion contains several paragraphs that extend beyond the scope of the results. Examples include extensive speculation about extremophiles, educational programs and student participation and environmental pollution pathways. These sections dilute the scientific focus of the manuscript. The discussion should be condensed and focused on the microbiome findings. Figure 2: The stacked bar plot is very crowded. Consider summarizing at class level rather than phylum level. Figure 3: Axis labels should include variance explained if available. Symbols representing habitats are difficult to distinguish. Several bacterial genera appear to be misspelled.

The manuscript addresses an interesting topic and presents a potentially valuable dataset. However, significant revisions are required to improve methodological clarity, correct inconsistencies, and strengthen the statistical analyses before the study can be considered for publication.

**********

6. PLOS authors have the option to publish the peer review history of their article (what does this mean?). If published, this will include your full peer review and any attached files.

If you choose “no”, your identity will remain anonymous but your review may still be made public.

**Do you want your identity to be public for this peer review?**  For information about this choice, including consent withdrawal, please see our Privacy Policy.

Reviewer #1: **Yes:** Meiling Hong, College of Life Science, Hainan Normal University

Reviewer #2: No

**********

[NOTE: If reviewer comments were submitted as an attachment file, they will be attached to this email and accessible via the submission site. Please log into your account, locate the manuscript record, and check for the action link "View Attachments". If this link does not appear, there are no attachment files.]

To ensure your figures meet our technical requirements, please review our figure guidelines: https://journals.plos.org/plosone/s/figures

You may also use PLOS’s free figure tool, NAAS, to help you prepare publication quality figures: https://journals.plos.org/plosone/s/figures#loc-tools-for-figure-preparation.

NAAS will assess whether your figures meet our technical requirements by comparing each figure against our figure specifications.

# Author comment: Author response to Decision Letter 1

**DOI:** 10.1371/journal.pone.0353172.r002

11 Jun 2026

We thank the reviewers for their insightful comments, which have greatly improved our manuscript. Below, we provide a point-by-point response to these comments, our efforts to incorporate them into the current manuscript, and, at times, our reasoning for not incorporating them (line numbers refer to tracked changes of version).

Referee: 1

This manuscript presents a timely and well-structured investigation into the microbial communities associated with an invasive freshwater turtle, the red-eared slider, across urban habitats in Austin, Texas. The study employs 16S rRNA amplicon sequencing to characterize microbial profiles from five body sites and compares them across several urban ponds/lakes. The topic is relevant to microbial ecology, invasion of biology, and public health, and the inclusion of student researchers adds an educational dimension to the work. While the core findings are clear and well-supported by the data, several methodological and interpretational aspects could be strengthened to enhance the manuscript's impact and clarity. But there are still some major concerns and suggestions:

We thank reviewer 1 for their positive comments and we incorporated their suggestions to improve our study.

1.The introduction effectively reviews sea turtle microbiomes but could briefly reference key freshwater turtle microbiome studies beyond the red-eared slider to better contextualize the work.

Agreed. We have added lines on freshwater turtle microbiome studies beyond red-eared sliders (line 80-87)

2.The absence of water/sediment samples from each habitat limits the ability to distinguish between environmentally derived vs. host-specific microbes. In Discussion Section P303-305, the higher relative abundance of Cyanobacteria is relative to the environment. Additionally, future work should integrate paired host–environment sampling across habitats to disentangle these influences rigorously. Moreover, the temporal dimension is also overlooked.

We agree that the absence of paired environmental samples limits our ability to conclusively distinguish environmentally derived from host-specific microbes. In the revised Discussion (lines 315–317). We have explicitly acknowledged this limitation and clarified that our interpretations regarding the relative abundance of Cyanobacteria are made with caution due to the lack of direct environmental comparisons. We also point out that cloacal/oral samples are more likely to be indicative of gut microbiota, while those on the shell/skin are more likely influenced by the environment. We also point out that future studies should incorporate environmental sampling. Finally, we also now suggest that future studies should examine microbiomes across time/seasons to determine how temporal effects change microbiomes in different body regions (lines 403-409).

3. Sample size imbalance. Only two turtles were sampled from Lake Muller, compared to 10–14 from other sites. This may affect habitat-level comparisons and statistical power. Please justify this disparity or acknowledge it as a study limitation.

Fewer turtles were sampled from Lake Mueller because it was a larger body of water, making them harder to catch than at other sites. Rather than remove these samples from the analyses, we now have addressed these technical constraints in the methodology section, lines 155-156, acknowledging that a limited sample size from this habitat makes inferences about its population limited (lines 414-415).

4.The 16S rRNA approach provides community profiling but limits functional interpretation. A brief discussion on the potential metabolic roles of dominant taxa (e.g., Cyanobacteria on shells) would enrich the ecological narrative. If possible, mention whether any PICRUSt2 or similar inference was attempted, even if only preliminarily.

We thank the reviewer for the suggestion on using PiCRUSt2 for looking into metabolic roles of dominant taxa. In response, we did run PICRUSt. We found that potential metabolic profiles of the samples did vary based on body habitat, supporting our ASV diversity results (figure below). However, functional differences in microbiota were not the focus on this project. As our identification was only based on a small region of the 16S rRNA, we do not fully trust the metabolic inferences and prefer to not make them a focus of the current study. Therefore, we suggest a potential future project could use whole genome sequencing to look more convincingly at different metabolic profiles and functional diversity of turtle microbiomes.

5. The results are too simple. Some findings in discussion are not explained in the Result Sections.

We’re not sure what findings/results the reviewer is referring to but have addressed similar comments from the other reviewer that we hope address this comment. We do agree that our results are relatively simple, but do not see this as a limitation. We have made sure that the Results and the Discussion sections are more synced.

6. Pathogen discussion could be contextualized. While potential pathogens (e.g., Acinetobacter, Mycobacterium) are noted, their low abundance is appropriately highlighted. However, the public health implications could be better framed by referencing local or national guidelines on zoonotic risk from handled turtles.

We have softened the claims and added line 361-366 on future research of pathogen screening and added context on the zoonotic risk of handling turtles.

7. The discussion on extremophiles (e.g., Chloroflexi, Halobacteriota) is interesting but somewhat speculative. Consider toning down claims about “behavior facilitating colonization” unless direct evidence or citations are provided.

We have removed the claims about behavior facilitating colonization and the excess discussion on extremophiles.

Referee 2:

This manuscript investigates the microbiome associated with different body regions of the invasive red-eared slider turtle (Trachemys scripta elegans) across several urban freshwater habitats using 16S rRNA sequencing. The study addresses an interesting and relevant topic in microbial ecology, invasive species biology, and urban ecosystem research. The dataset includes multiple body locations (carapace, plastron, skin, oral cavity, cloaca) and several sampling sites, which are valuable for understanding microbiome structure in reptiles.

However, the manuscript requires substantial revision before it can be considered for publication.

Several methodological aspects are insufficiently explained; statistical analyses need clarification, and parts of the discussion overinterpret the results. In addition, the manuscript contains inconsistencies in sampling description, analytical procedures, and interpretation of microbiome data.

Overall, the study has potential, but major revisions are necessary to improve methodological transparency, strengthen statistical analyses, and better align conclusions with the presented data.

We thank the reviewer for their useful comments and the manuscript improved by them.

The abstract states that: “community composition strongly structured by body location rather than habitat.” However, PERMANOVA results show that both factors were significant. This should be clarified.

We thank the reviewer for this observation. We have revised the abstract to clarify that both body location and habitat of origin significantly influenced microbial community composition.

The description of sampling sites and sampling dates is inconsistent and confusing. For example, in the methods section, sampling is reported to occur between September 2023 and May 2024, yet Lake Muller samples are reported from April 2025 (page ~13). The study refers to five locations, but the site descriptions and sample counts are difficult to follow. A table summarizing the sampling effort should be added. This will greatly improve clarity.

We thank the reviewer for pointing out these inconsistencies, which mainly stemmed from some typos and similar errors when preparing the manuscript. Sampling occurred between September 2023 and May 2024; the reference to April 2025 for the Lake Muller samples was a typo, which has been corrected on line 155. To further improve clarity, we have added a table (Table S1) summarizing the sampling effort in the supplemental information to strengthen clarity for the readers.

The study includes two individuals of Pseudemys texana among the samples (Fig. 2 captions). This raises several issues: Why were individuals from different species included in the microbiome analysis? Were they included in statistical analysis? If so, how was species identity controlled? Because microbiomes are often host-species specific, mixing species may bias the results. The authors should either: remove these samples from the analysis or clearly justify their inclusion and statistically control for species effects.

Individuals of Pseudemys texana were collected opportunistically. Given the lack of prior microbiome data for this species, we included these samples to provide preliminary reference data and contribute to publicly available datasets.

We agree that differences in host species can influence microbiome composition. To account for this, species identity was included as a factor in the PERMANOVA models. Species did not explain a significant portion of variation in microbial community composition (R² = 0.003, p = 0.920), indicating that inclusion of these samples did not bias the overall results, although we acknowledge a more focused study with larger sample sizes for P. texana would be needed to appropriately test this.

We acknowledge that the comparison is highly unbalanced (n = 139 vs. n = 3 samples) and therefore underpowered to detect species-level effects. We have clarified this limitation in the revised manuscript and justified including these samples to aid future work (lines 317-318). We have also changed the figures to make it clear which samples are from P. texana.

The authors state that rarefaction depth was set to 600,000 reads (Methods section). However: The average read count per sample is ~779,776 reads. Rarefaction at such a high depth may remove a large proportion of samples. The manuscript must clarify: How many samples were retained after rarefaction, whether rarefaction curves justified this threshold, and whether lower rarefaction depths were tested.

We thank the reviewer for pointing out this inconsistency. The originally reported rarefaction depth of 600,000 reads was incorrect and has been revised in the manuscript. Following DADA2 quality filtering and denoising, the number of usable reads per sample was substantially reduced relative to the initial sequencing depth. Alpha rarefaction analyses demonstrated that diversity plateaued between approximately 100,000–150,000 reads (Fig. S2), indicating that sequencing depth was sufficient to capture most microbial diversity across samples. Based on these results, analyses were conducted using a rarefaction depth of 150,000 reads. We have clarified this in the revised Methods section and updated the manuscript accordingly (Lines 215-216).

The authors filtered ASVs to those: “present in at least 50 of 142 samples.” This threshold is extremely strict and will eliminate rare taxa, which can be ecologically important in microbiome studies. The authors should: justify this filtering strategy: evaluate whether it removed site- or body-specific taxa, report the number of ASVs before and after filtering.

We agree that the threshold was overly strict, so we changed the filtering criterion to ASVs present in at least 5 of 142 samples. This increased the number of retained ASVs, and the numbers before and after filtering are now included in Supplemental Table 2. Importantly, this change did not substantially alter the overall results or interpretations of the study.

The manuscript reports that sex did not explain variation, yet the PERMANOVA result shows: F = 3.28, p = 0.001. A p-value of 0.001 indicates significance, contradicting the interpretation. This discrepancy must be corrected. PERMANOVA assumes homogeneous dispersion among groups. The manuscript does not report whether PERMDISP tests were conducted. Without this test, the interpretation of PERMANOVA results is incomplete. The NMDS stress value is 0.196. This value is relatively high and indicates a moderate representation of the data.

The originally reported PERMANOVA result for sex (p = 0.001) was based on a univariate model. We have now reanalyzed the data using a multivariable PERMANOVA including site and body location, which revealed that sex does not explain a significant portion of variation after accounting for these variables (PERMANOVA, R² = 0.006, p = 0.142). This indicates that the previously observed effect of sex was because it was tested on its own and not taking into consideration body location and habitat of origin. We have updated the manuscript accordingly.

In addition, based on the reviewer’s comment we conducted PERMDISP analyses to assess homogeneity of dispersion among groups. No significant differences in dispersion were detected for body location, habitat, or sex (all p > 0.05), confirming that PERMANOVA results reflect true compositional differences rather than differences in group variance. These results are now reported in the revised manuscript (lines 310-313).

The authors should: report whether additional dimensions were tested and discuss limitations of the ordination. The discussion repeatedly suggests that turtle microbiomes reflect environmental microbial communities. However: “we did not include environmental water samples.” This severely limits interpretation. Without environmental samples, claims about environmental influence remain speculative. The authors should: explicitly acknowledge this limitation and reduce speculation regarding environmental microbial sources.

We agree and we have included our limitation from not having environmental samples and we have reduced the speculation regarding environmental microbial resources (lines 319-322). Additional ordination dimensions were evaluated using a 3-dimensional NMDS. The 3-dimensional solution reduced stress from 0.196 to 0.141, indicating improved representation of community dissimilarities. However, the 2-dimensional NMDS was retained for visualization purposes and ease of interpretation.

The manuscript identifies genera containing pathogens such as: Acinetobacter, Mycobacterium and Roseomonas, however: 16S rRNA sequencing cannot identify pathogenic strains; many species in these genera are harmless environmental microbes. Therefore, statements suggesting public health risk should be softened unless supported by targeted pathogen screening.

We agreed that many of these genera are harmless environmental microbes, and we have softened the claims and added a line on future research of pathogen screening (lines 392-394). We also note this comment underlies our response to R1 above about how only limited functional inference can be made based on 16S data.

The discussion contains several paragraphs that extend beyond the scope of the results. Examples include extensive speculation about extremophiles, educational programs and student participation, and environmental pollution pathways. These sections dilute the scientific focus of the manuscript. The discussion should be condensed and focused on microbiome findings.

We feel some speculation in the Discussion is warranted to spur future research, but also agree with the reviewer that too much speculation may dilute the scientific merit of the manuscript. We therefore removed the discussion on extremophiles from the manuscript (also in accordance with R1’s comments). However, as a major point of this work was to develop an undergraduate-based research program on biodiversity, we feel it is important to include this perspective in the manuscript. To make this clearer, we have included more information to the introduction of the manuscript to highlight the potential role of urban ponds in student-led research and the motivation behind our work (line 131-134).

Figure 2: The stacked bar plot is very crowded. Consider summarizing class level rather than phylum level.

We are unsure about

Attachment Submitted filename: R2R_26-01884_Turtle_paper_MCO_JCH_060126.docx

# Editor report: Decision Letter 1

**DOI:** 10.1371/journal.pone.0353172.r003

**Authors:** Murtada D. Naser

21 Jun 2026

Comparative Analysis of the Mucosal and Shell Microbiota of Trachemys scripta elegans Across Multiple Urban Freshwater Habitats

PONE-D-26-01884R1

Dear Dr. Mariangel Correa Orellana,

We’re pleased to inform you that your manuscript has been judged scientifically suitable for publication and will be formally accepted for publication once it meets all outstanding technical requirements.

Within one week, you’ll receive an e-mail detailing the required amendments. When these have been addressed, you’ll receive a formal acceptance letter and your manuscript will be scheduled for publication.

An invoice will be generated when your article is formally accepted. Please note, if your institution has a publishing partnership with PLOS and your article meets the relevant criteria, all or part of your publication costs will be covered. Please make sure your user information is up-to-date by logging into Editorial Manager at Editorial Manager® and clicking the ‘Update My Information' link at the top of the page. For questions related to billing, please contact billing support.

If your institution or institutions have a press office, please notify them about your upcoming paper to help maximize its impact. If they’ll be preparing press materials, please inform our press team as soon as possible -- no later than 48 hours after receiving the formal acceptance. Your manuscript will remain under strict press embargo until 2 pm Eastern Time on the date of publication. For more information, please contact onepress@plos.org.

Kind regards,

Murtada D. Naser

Academic Editor

PLOS One

Additional Editor Comments (optional):

Reviewers' comments:

# Editor report: Acceptance letter

**DOI:** 10.1371/journal.pone.0353172.r004

**Authors:** Murtada D. Naser

PONE-D-26-01884R1

PLOS One

Dear Dr. Correa Orellana,

I'm pleased to inform you that your manuscript has been deemed suitable for publication in PLOS One. Congratulations! Your manuscript is now being handed over to our production team.

At this stage, our production department will prepare your paper for publication. This includes ensuring the following:

* All references, tables, and figures are properly cited

* All relevant supporting information is included in the manuscript submission,

* There are no issues that prevent the paper from being properly typeset

You will receive further instructions from the production team, including instructions on how to review your proof when it is ready. Please keep in mind that we are working through a large volume of accepted articles, so please give us a few days to review your paper and let you know the next and final steps.

Lastly, if your institution or institutions have a press office, please let them know about your upcoming paper now to help maximize its impact. If they'll be preparing press materials, please inform our press team within the next 48 hours. Your manuscript will remain under strict press embargo until 2 pm Eastern Time on the date of publication. For more information, please contact onepress@plos.org.

You will receive an invoice from PLOS for your publication fee after your manuscript has reached the completed accept phase. If you receive an email requesting payment before acceptance or for any other service, this may be a phishing scheme. Learn how to identify phishing emails and protect your accounts at https://explore.plos.org/phishing.

If we can help with anything else, please email us at customercare@plos.org.

Thank you for submitting your work to PLOS ONE and supporting open access.

Kind regards,

PLOS ONE Editorial Office Staff

on behalf of

Dr. Murtada D. Naser

Academic Editor

PLOS One
